# Dietary magnesium deficiency impaired intestinal structural integrity in grass carp (*Ctenopharyngodon idella*)

**DOI:** 10.1038/s41598-018-30485-8

**Published:** 2018-08-23

**Authors:** Shuo-Peng Wei, Wei-Dan Jiang, Pei Wu, Yang Liu, Yun-Yun Zeng, Jun Jiang, Sheng-Yao Kuang, Ling Tang, Yong-An Zhang, Xiao-Qiu Zhou, Lin Feng

**Affiliations:** 1 Animal Nutrition Institute, Sichuan Agricultural University, Chengdu, 611130 China; 2 Fish Nutrition and safety Production University Key Laboratory of Sichuan Province, Sichuan Agricultural University, Chengdu, 611130 China; 3 Key Laboratory for Animal Disease-Resistance Nutrition of China Ministry of Education, Sichuan Agricultural University, Chengdu, 611130 China; 4 Animal Nutrition Institute, Sichuan Academy of Animal Science, Chengdu, 610066 China; 5 Institute of Hydrobiology, Chinese Academy of Sciences, Wuhan, 430072 China

## Abstract

Grass carp (223.85–757.33 g) were fed diets supplemented with magnesium (73.54–1054.53 mg/kg) for 60 days to explore the impacts of magnesium deficiency on the growth and intestinal structural integrity of the fish. The results demonstrated that magnesium deficiency suppressed the growth and damaged the intestinal structural integrity of the fish. We first demonstrated that magnesium is partly involved in (1) attenuating antioxidant ability by suppressing Nrf2 signalling to decrease antioxidant enzyme mRNA levels and activities (except *CuZnSOD* mRNA levels and activities); (2) aggravating apoptosis by activating JNK (not p38MAPK) signalling to upregulate proapoptotic protein (*Apaf*-*1*, *Bax* and *FasL*) and *caspase*-*2*, -*3*, -*7*, -*8* and -*9* gene expression but downregulate antiapoptotic protein (*Bcl*-*2*, *IAP* and *Mcl*-*1b*) gene expression; (3) weakening the function of tight junctional complexes (TJs) by promoting myosin light chain kinase (MLCK) signalling to downregulate TJ gene expression [except *claudin*-*7*, *ZO*-*2b* and *claudin*-*15* gene expression]. Additionally, based on percent weight gain (PWG), against reactive oxygen species (ROS), against *caspase*-*9* and *claudin*-*3c* in grass carp, the optimal dietary magnesium levels were calculated to be 770.38, 839.86, 856.79 and 811.49 mg/kg, respectively.

## Introduction

Magnesium is an essential element well known for its role in activating enzymes for nutrition metabolism, energy metabolism and nucleic acid biochemistry in mammals^1^. Emerging evidence has revealed that magnesium deficiency could induce inflammation in human^2^ and rat intestines^3^. A recent study demonstrated that inflammation could impair animal intestinal structural integrity^4^. These results indicate that magnesium deficiency might impair animal intestinal structural integrity. Unfortunately, so far, only one study has observed that magnesium deficiency impaired mouse intestinal structural integrity by down-regulating *occludin* and *ZO*-*1* gene expression^5^. However, this research still lacks a systematic approach to animal intestinal structural integrity, and it did not investigate the underlying mechanisms. Therefore, it is imperative to explore the effects of magnesium deficiency on intestinal structural integrity and to conduct deeper examination on the molecular mechanisms in animals.

In fish, intestinal structural integrity is influenced by cellular structural integrity, which can be impaired by cell apoptosis and oxidative damage^6^. Chen *et al*.^7^ found that antioxidants could attenuate oxidative damage in grass carp intestine. Moreover, another study observed that cell apoptosis depended on apoptosis -related proteins of the caspase family (caspase-2, -3, -7, -8 and -9) in mammals^8^. In fish, antioxidants and apoptosis-related proteins are deeply dependent on regulation by Nrf2^9^ and JNK^10^, respectively. So far, the fragmentary research of oxidative damage (only detecting MDA and ROS) and cell apoptosis (only detecting caspase-3) in animals has been focused on the liver, kidney, heart, brain, muscle, thymus and spleen^11–14^. However, animal intestines differ from these tissues and organs in terms of oxidative damage and cell apoptosis, and here, we list some of the differences. First, during normal aging of both humans and animals, some postmitotic tissues can be renewed by cell apoptosis in these tissues and organs (such as brain, muscle, heart and liver)^15,16^. In the animal intestine, cell apoptosis takes place only in limited areas or cells (crypt, early transit cells and villus tip)^17^. Second, one study reported that the different lipid components could induce different degrees of oxidative damage in fish^18^. The metabolism of lipids is different between animal intestines and other organs. It was reported that the animal intestine is another independent organ, second to the liver, that metabolizes lipids in the animal body^19^. However, there also exist some differences between the intestine and liver in lipid metabolism in animals. To our knowledge, it has been demonstrated that magnesium could decrease the glucagon content in the dog pancreas^20^, which could inhibit lipid synthesis in the animal liver (rather than in the animal intestine)^19^. Additionally, in animal livers, magnesium could activate the phosphatidylethanolamine *N*-methyltransferase pathway^21,22^ to synthesize lecithin (an important lipid in the cytomembrane) in the liver (rather than in the intestine)^23^. This evidence indicates that the effect of magnesium on the structural integrity of animal intestines is different from that in other organs. However, to date, there have been no studies on animal intestines focused on the relationship between magnesium deficiency and oxidation, antioxidants and cell apoptosis, and no reports have addressed the corresponding mechanisms in animals. In rat plasma, magnesium deficiency could decrease the phosphorus content^24^. Previously, our laboratory found that phosphorus deficiency downregulated *Nrf2* gene expression in grass carp skin^25^. Additionally, Hsu JM and Smith JJ showed that magnesium deficiency depressed ascorbic acid synthesis in the rat liver^26^, and depressed levels of ascorbic acid could aggravate human colon cancer cell apoptosis^27^. In rat serum, magnesium deficiency could elevate the mRNA level of *IL*-*1β*^28^, which could upregulate *caspase*-*2*, -*8* and -*9* gene expression in human foetal membranes^29^. Additionally, a study showed that magnesium deficiency increased the content of arachidonic acid (AA) in rat renal epithelial cell^30^, which could enhance the JNK protein content in human monocytes^31^. Hence, it is imperative to explore the potential relationship between magnesium deficiency and antioxidants, oxidation, and cell apoptosis as well as the corresponding mechanisms in animal intestines.

Apart from cellular structural integrity, intercellular structural integrity also takes part in maintaining fish intestinal structural integrity^32^. As is known, intercellular structural integrity is related to TJs (such as claudins and ZO-1) in pig intestines^33^, which are under the control of MLCK in the bovine brain^34^. Unfortunately, only scarce evidence is available about the relationship between magnesium deficiency and TJs (except occludin and ZO-1) as well as the underlying mechanisms in animals. Studies have demonstrated that magnesium deficiency enhanced *TNF*-*α* gene expression in human serum^35^ and IFN-γ secretion in rat blood^36^. In human colonic epithelial cells, co-treatment with TNF-α and IFN-γ could decrease the claudin-3 protein level^37^. Furthermore, Song *et al*.^38^ reported that magnesium deficiency could increase the insulin content in human plasma. In the 3T3-L1 adipocytes of rats, insulin could stimulate phosphorylation of MLCK^39^. According to these discoveries, it is imperative to systematically investigate the relationship between magnesium deficiency and TJs as well as the corresponding molecular mechanisms in animals.

One of the most widely cultured freshwater fish in the world is the grass carp^40^. To date, information on magnesium requirements has only focused on juvenile grass carp and was based only on the PWG^41^. Nevertheless, fish in different indices^42^ and different growth stages^43,44^ have different nutrients requirements. Hence, studying the dietary magnesium requirements of grass carp (223.85–757.33 g) is imperative.

In our current study, apart from systematic research on the relationship between magnesium deficiency and TJs, we innovatively investigated the relationship between magnesium deficiency and oxidation, antioxidants, and cell apoptosis as well as the corresponding signalling molecules (*Nrf2*, *MLCK* and *JNK*) in animal intestines, aiming to determine the possible mechanism of fish intestinal structural integrity with magnesium treatment. Meanwhile, the magnesium requirements of grass carp (223.85–757.33 g) were studied, which could provide practical evidence and references for commercial feed formulation in this fish.

## Results

### Growth performance

**Figure 1:**
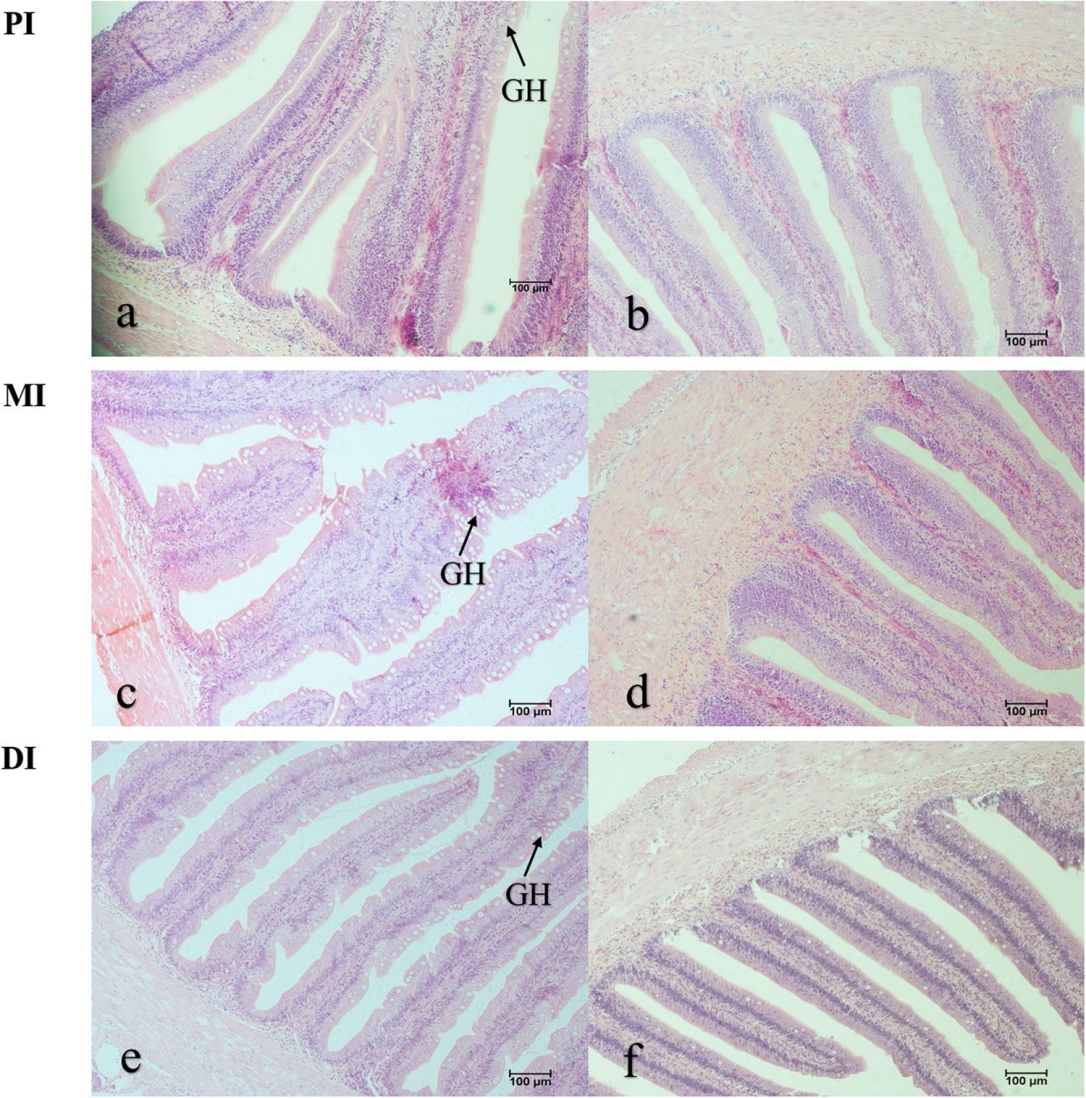
The histology of PI, MI and DI of grass carp fed diets containing graded levels of magnesium. The magnesium deficiency group (**a**,**c**,**e**), the optimal magnesium group (**b**,**d**,**f**). Arrowhead showed goblet cell hyperplasia (GH). Magnesium deficiency group: 73.54 mg/kg group. Optimal magnesium group: 861.67 mg/kg group.

As our data shows in Table 1, PWG, FBW and SGR all increased as the magnesium level rose to 861.67 mg/kg, and decreased significantly (*P* < 0.05). Fish fed magnesium at 861.67 mg/kg showed the highest FE compared to other groups. Additionally, when the magnesium level rose to 691.55, 861.67, 861.67 and 861.67 mg/kg, respectively, ILI, IW, ISI and IL all increased and thereafter decreased sharply (*P* < 0.05). Moreover, FI increased dramatically as the magnesium level rose to 691.55 mg/kg (*P* < 0.05), decreasing thereafter. Compared with the optimal-magnesium group, the magnesium-deficient group showed a significant decrease in the Na^+^, K^+^-ATPase and AKP activities of grass carp intestines as well as the magnesium concentrations in grass carp intestines and serum (*P* < 0.05). Grass carp fed a magnesium-deficient diet exhibited goblet cell hyperplasia in the intestines (Fig. 1). This phenomenon should be deeply investigated.

**Table 1:** Growth performance, intestinal length (cm/fish), intestinal weight (g/fish), intestinal length index (%), intestinal somatic index (%) and related sensitive indices in grass carp (*Ctenopharyngodon idellus*) fed diets containing graded levels of magnesium for 60 days.

Magnesium (mg/kg)	73.54		281.37		487.49		691.55		861.67		1054.53	
	Mean	SD	Mean	SD	Mean	SD	Mean	SD	Mean	SD	Mean	SD
IBW*	224.22^a^	0.38	223.56^a^	0.38	223.78^a^	0.38	223.56^a^	0.38	224.00^a^	0.67	224.00^a^	0.67
FBW*	668.17^a^	10.80	696.87^b^	11.38	731.67^c^	15.28	753.30^d^	7.77	757.33^d^	11.75	730.13^c^	10.00
PWG*	198.00^a^	5.04	211.73^b^	5.39	226.97^c^	7.26	236.96^d^	3.04	238.10^d^	5.82	225.96^c^	5.02
SGR*	1.82^a^	0.03	1.89^b^	0.03	1.97^c^	0.04	2.02^d^	0.02	2.03^d^	0.03	1.97^c^	0.03
FI*	672.01^a^	8.17	697.80^b^	3.37	726.01^c^	1.82	738.74^d^	1.58	738.17^d^	1.50	725.67^c^	3.28
FE*	66.06^a^	0.98	67.83^a,b^	1.89	69.96^b,c^	2.21	71.71^c^	1.01	72.25^c^	1.56	69.75^b,c^	1.37
IL^†^	52.34^a^	3.24	58.82^b^	2.96	58.57^b^	3.66	60.93^b^	6.09	60.40^b^	4.24	53.33^a^	5.33
IW^†^	10.78^a^	0.92	11.70^a,b^	0.64	12.81^c^	0.83	14.10^d^	1.32	14.17^d^	1.02	12.04^b,c^	1.12
ILI^†^	161.07^a^	7.01	173.90^b^	9.26	175.99^b^	11.47	178.77^b^	18.65	179.20^b^	12.55	153.69^a^	13.00
ISI^†^	1.58^a^	0.16	1.64^a^	0.15	1.79^b^	0.12	1.85^b^	0.17	1.89^b^	0.18	1.63^a^	0.17
**Na** ^**+**^ **/K** ^**+**^ **-ATPase activities (μmol of phosphorus released g/tissue per h)**												
PI^※^	75.19^a^	7.40	87.37^b^	8.65	99.85^c^	4.23	108.20^c,d^	8.62	109.65^d^	10.26	113.90^d^	3.46
MI^※^	55.26^a^	5.06	65.25^b^	5.34	72.98^c^	6.06	81.37^d^	6.47	84.58^d^	8.39	87.11^d^	5.44
DI^※^	64.17^a^	3.03	79.75^b^	5.40	89.34^c^	7.14	90.49^c^	8.52	91.78^c^	5.95	93.56^c^	8.65
**AKP (mmol of nitrophenol released g/tissue per h)**												
PI^※^	63.69^a^	3.88	72.50^b^	5.74	82.93^c^	7.06	81.57^c^	6.78	80.82^c^	4.16	83.39^c^	7.55
MI^※^	39.92^a^	3.07	47.13^b^	4.65	54.44^c^	3.58	61.75^d^	3.29	60.11^d^	4.32	62.94^d^	2.13
DI^※^	37.62^a^	3.43	46.69^b^	4.35	52.90^c^	5.27	52.53^c^	2.57	56.81^c^	4.99	52.26^c^	4.25
Serum magnesium concentration (mmol/l)*												
	0.71^a^	0.04	0.96^b^	0.09	1.26^c^	0.09	1.55^d^	0.15	1.62^d^	0.05	1.60^d^	0.06
**Intestinal magnesium concentration (mg/kg)**												
PI^※^	72.38^a^	3.06	90.17^b^	5.98	114.82^c^	6.35	128.47^d^	11.11	130.21^d^	10.83	132.00^d^	12.18
MI^※^	40.19^a^	2.86	60.40^b^	3.36	72.07^c^	1.97	95.61^d^	5.72	101.68^e^	4.87	102.95^e^	6.70
DI^※^	11.39^a^	0.84	19.67^b^	1.12	24.02^c^	2.21	24.65^c^	2.28	23.57^c^	2.00	25.29^c^	2.49

*Values are means and standard deviations of three replicate groups, with 30 fish in each group. ^a,b,c,d^Mean values within a row with unlike superscript letters are significantly different (*P* < 0.05; ANOVA and Duncan’s multiple range tests). IBW, initial body weight (g/fish); FBW, final body weight (g/fish); PWG, percent weight gain (%); SGR, specific growth rate (%/day); FI, feed intake (g/fish); FE, feed efficiency (%); AKP: alkaline phosphatase (mmol of nitrophenol released g/protein per h); Na+ /K+ -ATPase: (μmol of phosphorus released g/protein per h).

^†^Values are means and standard deviations of nine replicates. ^a,b,c,d^Mean values within a row with unlike superscript letters are significantly different (*P* < 0.05; ANOVA and Duncan’s multiple range tests). IL, intestinal length (cm); IW, intestinal weight (g/fish); ILI, intestinal length index (%); ISI, intestinal somatic index (%).

^※^Values are means and standard deviations of six replicates. ^a,b,c,d^Mean values within a row with unlike superscript letters are significantly different (*P* < 0.05; ANOVA and Duncan’s multiple range tests).

### Oxidative stress parameters in the intestines of grass carp

In Table 2, we can clearly find the effects on the antioxidant related substances in grass carp intestines with magnesium treatment. The content of MDA in grass carp intestines decreased as the magnesium level rose to 861.67 mg/kg and increased significantly afterward (*P* < 0.05). Meanwhile, the highest PC contents were observed in intestines of grass carp fed a dietary magnesium level of 73.54 mg/kg. Additionally, the ROS content decreased to the lowest level in grass carp MI and PI as the magnesium levels rose to 861.67 mg/kg and in the DI of this fish as the magnesium level rose to 691.55 mg/kg, increasing dramatically thereafter (*P* < 0.05). On the contrary, the GPx and GST activities increased significantly (*P* < 0.05) in grass carp intestines when the magnesium levels rose to 861.67 mg/kg and thereafter decreased significantly (*P* < 0.05). The highest ASA activities were found in the PI of grass carp fed a dietary magnesium level of 861.67 mg/kg and in the DI and MI of this fish fed a dietary magnesium level of 691.55 mg/kg. The AHR activities and GSH contents increased significantly in grass carp MI and PI as the magnesium level rose to 691.55 mg/kg and in the DI of this fish as the magnesium level rose to 861.67 mg/kg (*P* < 0.05), thereafter decreasing. Additionally, fish fed a dietary magnesium level of 861.67 mg/kg showed maximum activities of T-SOD and MnSOD in grass carp intestines. Fish fed dietary magnesium levels of 861.67 mg/kg and 691.55 mg/kg showed the maximum CAT activities in the MI and in the DI and PI, respectively. The GR activities increased significantly (*P* < 0.05) in grass carp MI and PI at a magnesium level of 861.67 mg/kg, and in the DI of this fish at a magnesium level of 691.55 mg/kg, thereafter decreasing significantly (*P* < 0.05). Interestingly, magnesium did not influence the CuZnSOD activities in grass carp intestines.

**Table 2:** Effects of dietary magnesium on antioxidant related parameters in the PI, MI and DI of grass carp^※^.

Magnesium level (mg/kg)	73.54		281.37		487.49		691.55		861.67		1054.53	
	Mean	SD	Mean	SD	Mean	SD	Mean	SD	Mean	SD	Mean	SD
**PI**												
MDA	32.38^c^	1.27	21.87^b^	2.32	19.75^b^	1.87	17.45^a^	1.95	16.40^a^	1.43	21.02^b^	1.81
PC	6.78^e^	0.55	4.78^d^	0.43	3.42^c^	0.25	2.17^a^	0.19	2.42^a^	0.19	2.96^b^	0.19
ROS	100.00^e^	9.11	82.99^d^	6.46	71.51^c^	6.01	50.21^a^	1.74	49.49^a^	3.12	62.27^b^	2.49
ASA	71.32^a^	6.17	80.57^a^	7.43	91.37^b^	8.24	108.42^c^	9.65	121.43^d^	8.17	109.80^c^	7.05
AHR	39.19^a^	2.51	43.59^b,c^	1.62	46.14^c^	2.71	50.42^d^	3.01	45.80^b,c^	1.64	43.02^b^	1.65
T-SOD	16.07^a^	0.57	18.29^b^	0.53	20.46^c^	0.85	21.03^c^	0.99	24.91^e^	0.74	22.02^d^	0.81
CuZnSOD	10.10^a^	0.56	10.58^a^	0.36	10.53^a^	0.42	10.13^a^	0.15	10.54^a^	0.45	10.49^a^	0.50
MnSOD	5.97^a^	0.41	7.71^b^	0.35	9.93^c^	0.63	10.90^d^	0.94	14.37^e^	0.69	11.53^d^	1.08
CAT	3.15^a^	0.23	3.20^a^	0.25	3.30^a^	0.31	3.65^b^	0.34	3.42^a,b^	0.12	3.25^a^	0.23
GPx	80.20^a^	7.34	98.06^b^	2.94	112.77^c^	8.10	127.72^d^	8.48	143.67^e^	6.84	118.54^c^	9.65
GST	35.66^a^	1.83	40.86^b^	3.44	43.54^b,c^	4.25	50.64^d^	4.46	59.23^e^	5.29	47.08^c,d^	4.02
GR	35.33^a^	3.06	41.07^b^	4.04	45.73^c,d^	3.50	46.69^d^	3.73	52.57^e^	3.29	41.98^b,c^	2.14
GSH	6.95^a^	0.40	7.86^b^	0.55	8.64^c,d^	0.76	9.98^e^	0.90	9.06^d^	0.18	8.08^b,c^	0.69
**MI**												
MDA	23.95^e^	2.08	19.90^d^	2.21	16.87^c^	1.28	14.75^b^	1.25	12.32^a^	1.12	15.04^b,c^	0.84
PC	9.08^d^	0.87	6.83^c^	0.57	6.24^c^	0.31	5.07^b^	0.36	3.94^a^	0.25	5.09^b^	0.38
ROS	100.00^e^	6.50	88.04^d^	6.42	80.43^c^	7.05	70.18^b^	5.64	59.57^a^	3.48	67.74^b^	5.85
ASA	80.25^a^	7.63	96.59^b^	4.63	114.09^c^	9.03	138.91^d^	13.85	123.21^c^	8.17	114.51^c^	11.29
AHR	55.61^a^	2.94	62.03^b,c^	5.21	66.87^c^	3.30	74.90^d^	4.45	64.30^b,c^	4.73	59.98^a,b^	2.65
T-SOD	12.98^a^	0.57	14.83^b^	0.61	16.31^c^	0.66	17.78^d^	0.38	18.68^d^	1.52	16.04^c^	1.39
CuZnSOD	7.73^a^	0.68	8.26^a^	0.55	8.21^a^	0.66	7.65^a^	0.45	7.91^a^	0.69	7.92^a^	0.78
MnSOD	5.25^a^	0.43	6.56^b^	0.38	8.10^c^	0.67	10.14^d^	0.29	10.77^d^	1.06	8.12^c^	0.78
CAT	2.58^a^	0.18	2.45^a^	0.19	2.62^a^	0.13	2.86^b^	0.15	2.89^b^	0.17	2.56^a^	0.23
GPx	80.98^a^	6.33	93.61^b^	7.51	120.33^c^	11.56	127.79^c^	9.85	140.71^d^	13.04	126.82^c^	6.66
GST	45.86^a^	4.07	52.37^b^	4.58	57.27^b,c^	5.11	62.36^c^	3.81	69.44^d^	4.66	62.22^c^	6.19
GR	30.59^a^	2.64	35.63^b^	2.77	40.22^c^	1.96	44.94^d^	1.66	52.43^e^	4.12	47.01^d^	3.72
GSH	6.70^a^	0.55	8.22^b^	0.77	9.96^c,d^	0.68	11.20^e^	0.63	10.53^d,e^	0.48	9.23^c^	0.92
**DI**												
MDA	25.78^d^	1.64	22.07^c^	1.01	19.09^b^	1.03	17.61^b^	1.39	13.92^a^	1.32	17.57^b^	1.70
PC	7.80^e^	0.61	6.95^d^	0.61	5.94^c^	0.31	5.18^b^	0.45	4.28^a^	0.21	5.30^b^	0.47
ROS	100.00^f^	7.35	84.24^e^	5.10	72.34^d^	2.08	41.19^a^	3.82	51.44^b^	4.52	64.20^c^	4.80
ASA	87.77^a^	8.04	99.50^b^	9.59	118.83^c,d^	9.17	135.06^e^	8.56	129.46^d,e^	11.48	115.57^c^	10.36
AHR	49.88^a^	3.78	56.37^b^	3.54	63.55^c^	6.24	70.22^d^	3.21	78.47^e^	7.55	70.90^d^	6.11
T-SOD	15.94^a^	0.72	16.53^a,b^	0.69	17.03^b,c^	0.44	17.59^c^	0.53	17.72^c^	0.65	17.37^c^	0.29
CuZnSOD	10.35^a^	0.74	10.11^a^	0.33	10.12^a^	0.50	9.95^a^	0.48	9.71^a^	0.46	10.17^a^	0.50
MnSOD	5.59^a^	0.47	6.42^b^	0.56	6.91^b,c^	0.20	7.63^d,e^	0.72	8.01^e^	0.34	7.20^c,d^	0.31
CAT	2.56^a^	0.20	2.52^a^	0.19	2.60^a^	0.15	2.86^b^	0.23	2.68^a,b^	0.13	2.54^a^	0.22
GPx	56.72^a^	4.96	69.26^b^	4.24	82.96^c^	7.87	88.70^c^	8.53	101.10^d^	6.18	89.75^c^	7.34
GST	62.11^a^	3.16	67.60^a^	6.48	75.16^b^	4.01	80.95^b,c^	3.85	85.15^c^	8.49	75.20^b^	5.53
GR	32.44^a^	2.00	36.85^b^	3.68	41.54^c^	2.21	53.49^e^	4.63	47.52^d^	3.72	44.92^c,d^	2.65
GSH	5.36^a^	0.35	6.30^b^	0.61	7.41^c^	0.41	8.67^d^	0.80	9.71^e^	0.82	8.62^d^	0.42

^※^Values are means and standard deviations of six replicates. ^a,b,c,d,e,f^Mean values within a row with unlike superscript letters are significantly different (*P* < 0.05). MDA, malondialdehyde (nmol/g tissues); PC, protein carbonyl (nmol/mg protein); ROS, reactive oxygen species (% DCF florescence); ASA, anti-superoxide anion (U/g protein); AHR, anti-hydroxyl radical (U/mg protein); T-SOD, total superoxide dismutase (U/mg protein); CuZnSOD (U/mg protein); MnSOD (U/mg protein); CAT (U/mg protein); GPx (U/mg protein); GST (U/mg protein); GR (U/g protein); GSH (mg/g protein).

**Figure 2:**
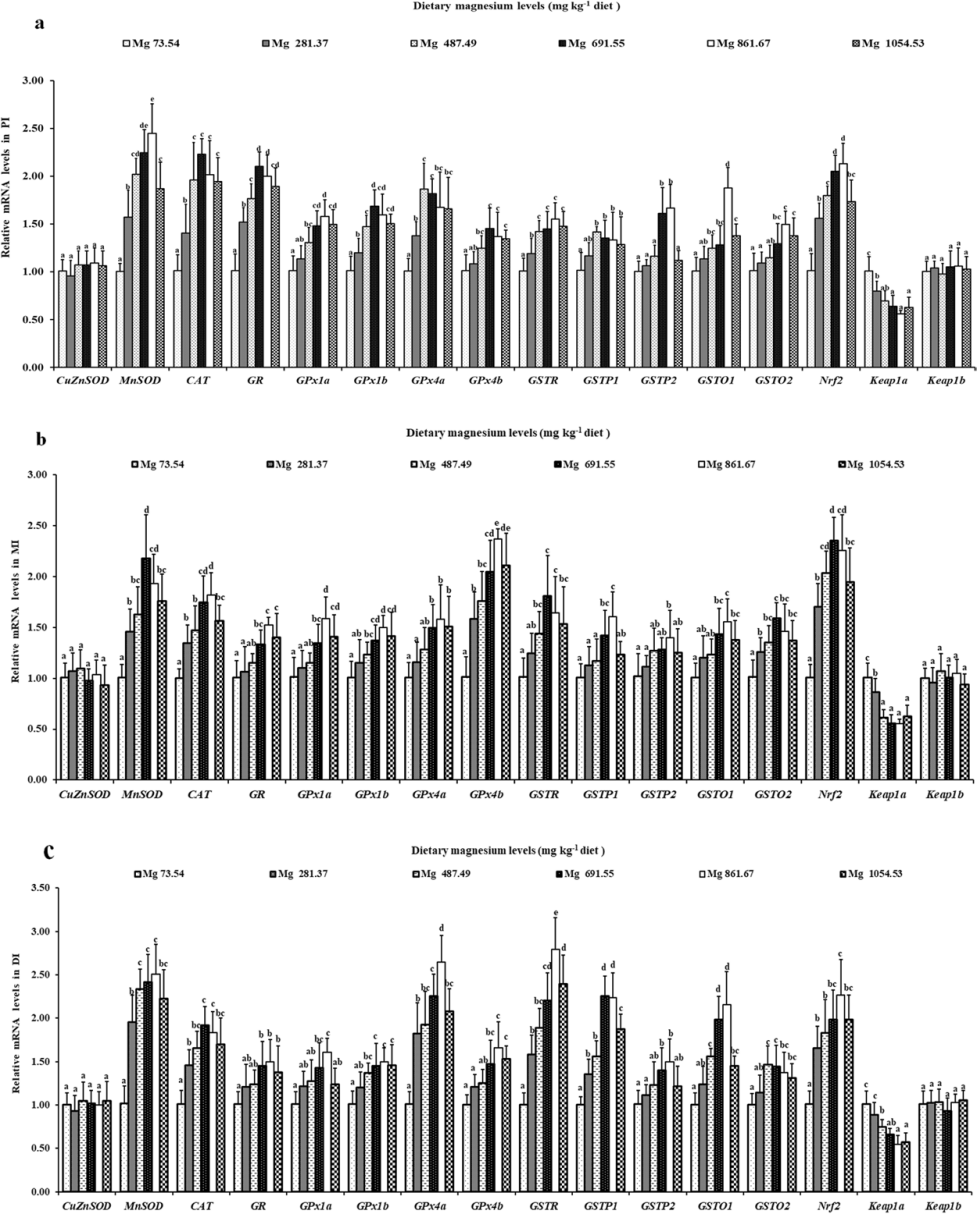
Relative gene expressions of antioxidant enzymes, *Nrf2*, *Keap1a and Keap1b* in PI (**a**), MI (**b**) and DI (**c**) of grass carp fed diets containing graded levels of magnesium. Data represent means of six fish in each group, error bars indicate S.D. Values having different letters are significantly different (*P* < 0.05; ANOVA and Duncan’s multiple range tests).

Observation of the effects on antioxidant-related gene expression also need mentioning (Fig. 2). The gene expression of *GSTP2*, *GPx1a* and *GSTO1* in grass carp intestines reached to a peak as the magnesium level rose to 861.67 mg/kg and then decreased. Furthermore, the gene expression of *GPx1b*, *GPx4b* and *GR* was upregulated under magnesium treatment with magnesium levels rose to 691.55 mg/kg in the PI and 861.67 mg/kg in the DI and MI and thereafter plateaued (*P* > 0.05). The *MnSOD*, *Nrf2* and *GSTR* gene expression was upregulated in grass carp DI and PI when fish received 861.67 mg/kg, and in the MI of this fish at a magnesium level of 691.55 mg/kg, decreasing afterwards. Simultaneously, the gene expression of other cytokines, namely, *CAT*, *GSTP1*, *GPx4a* and *GSTO2* was significantly higher in grass carp intestines in the optimal-magnesium group compared with the magnesium-deficient group (*P* < 0.05). In addition, the *Keap1a* gene expression in grass carp intestines decreased as the magnesium level rose to 861.67 mg/kg and plateaued thereafter (*P* > 0.05). Surprisingly, we found that dietary magnesium did not alter the *Keap1b* and *CuZnSOD* mRNA levels in grass carp intestines.

### Protein levels of Nrf2 in the intestines of grass carp

**Figure 3:**
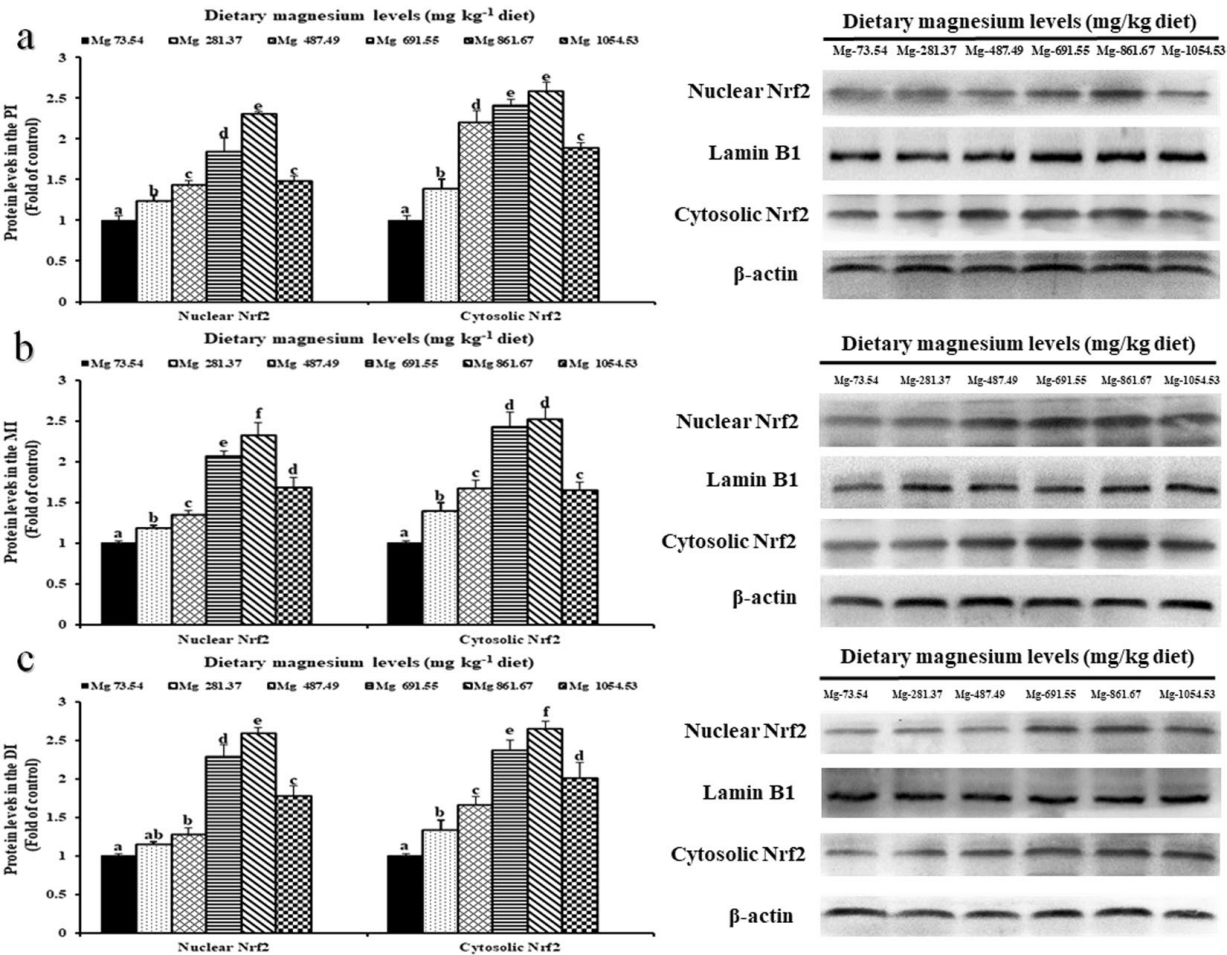
Western blot analysis of nuclear Nrf2 and cytosolic Nrf2 in the PI (**a**), MI (**b**) and DI (**c**) of grass carp fed diets containing graded levels of magnesium. Data represent means of three fish in each group, error bars indicate S.D. Values having different letters are significantly different (*P* < 0.05; ANOVA and Duncan’s multiple range test).

The impacts of magnesium on cytosolic Nrf2 and nuclear Nrf2 protein levels in grass carp intestines are shown in Fig. 3. When the magnesium level rose to 861.67 mg/kg, the protein levels of nuclear Nrf2 increased sharply (*P* < 0.05) in grass carp intestines and then declined significantly (*P* < 0.05). When the magnesium level rose to 861.67 mg/kg, increased protein levels of cytosolic Nrf2 were found in grass carp intestines, which then decreased significantly (*P* < 0.05).

### DNA fragmentation and mRNA levels of genes related to apoptosis in the intestines of grass carp

**Figure 4:**
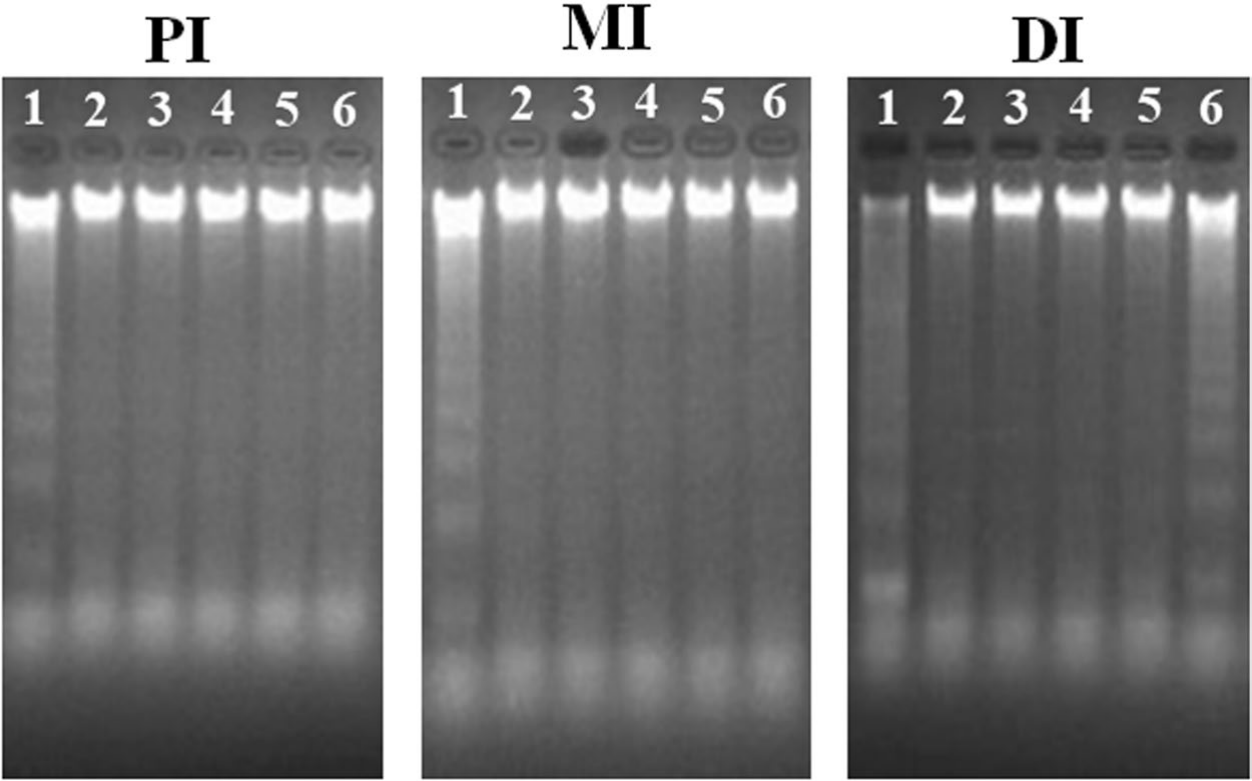
Effects of different dietary magnesium levels on DNA fragmentation in PI, MI and DI of grass carp using agarose gel electrophoresis. Lane 1: magnesium deficiency: 73.54 mg/kg. Lane 2–Lane 6: levels of dietary magnesium were 281.37, 487.49, 691.55, 861.67 and 1054.53 mg/kg, respectively. This experiment was repeated three times with similar results achieved.

**Figure 5:**
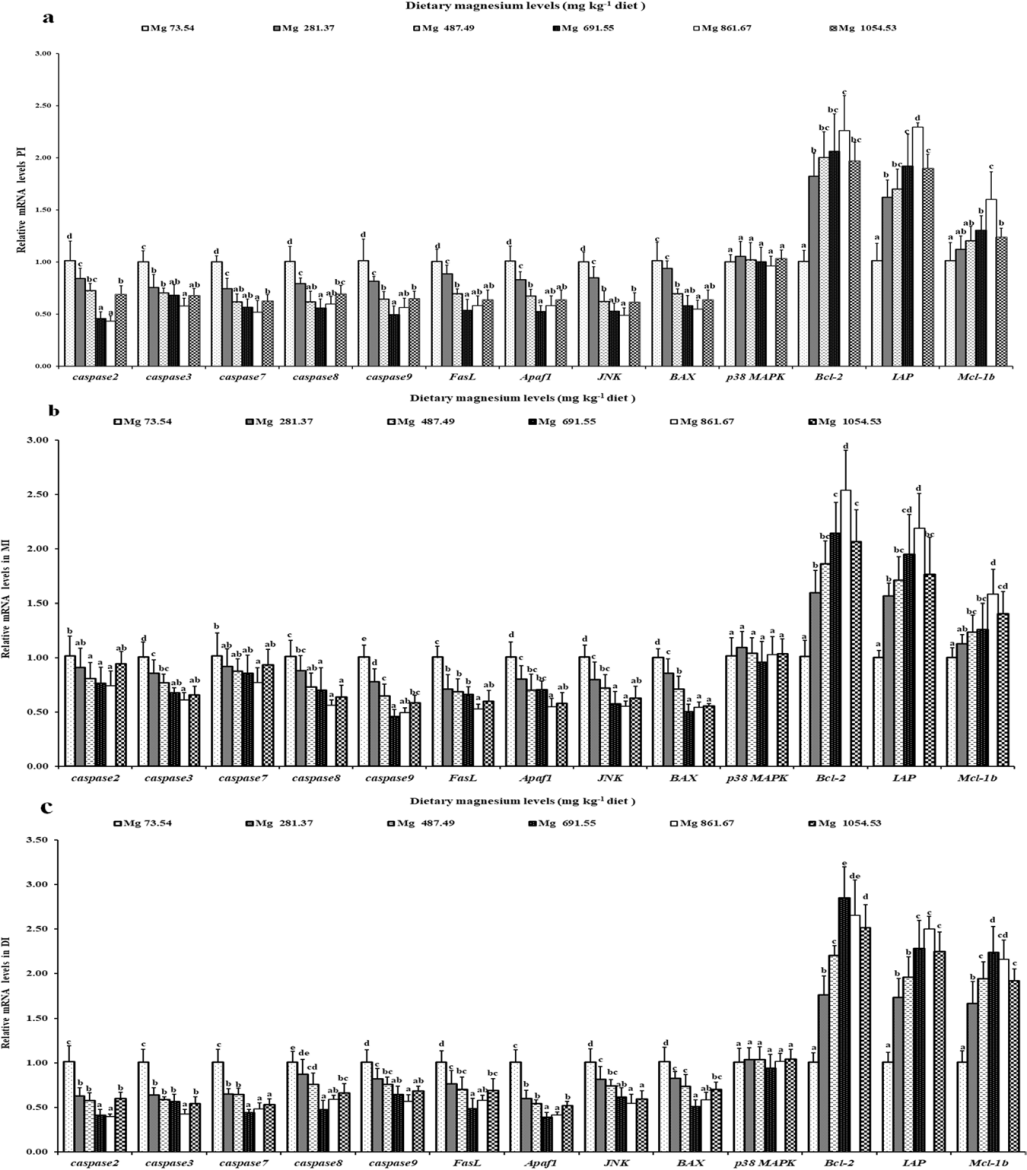
Relative gene expressions of apoptotic parameters in PI (**a**), MI (**b**) and DI (**c**) of grass carp fed diets containing graded levels of magnesium. Data represent means of six fish in each group, error bars indicate S.D. Values having different letters are significantly different (*P* < 0.05; ANOVA and Duncan’s multiple range tests).

DNA fragmentation results under magnesium treatment in grass carp intestines are shown in Fig. 4. Our current results indicated that a magnesium level of 73.54 mg/kg induced a ladder-like DNA fragment pattern in grass carp intestines. Cell apoptosis-related proteins in grass carp intestines were also affected by dietary magnesium. As our data show in Fig. 5, *FasL*, *caspase*-*8* and *Apaf*-*1* gene expression decreased as magnesium level rose to 691.55 mg/kg in grass carp DI and PI and to 861.67 mg/kg in the MI and then increased. Additionally, magnesium at a level of 861.67 mg/kg first suppressed and then enhanced the gene expression of *caspase*-*2*, -*3* and *JNK* in grass carp intestines. *Bax* gene expression was the lowest in grass carp PI when the magnesium level rose to 861.67 mg/kg and in the DI and MI at a magnesium level of 691.55 mg/kg. Fish fed a magnesium level of 73.54 mg/kg displayed the highest levels of *caspase*-*7* and -*9* gene expression in grass carp intestines among the six treatment groups. However, some cytokines showed different tendencies under magnesium treatment. The gene expression of inhibitor of apoptosis proteins (*IAP*) increased in grass carp intestines as the magnesium level rose to 861.67 mg/kg, thereafter decreasing. Simultaneously, the highest levels of *Bcl*-*2* and *Mcl*-*1b* gene expression were found in the PI and MI of grass carp fed a magnesium level of 861.67 mg/kg and in the DI of this fish fed a magnesium level of 691.55 mg/kg. Surprisingly, magnesium did not alter the gene expression of p38 mitogen activated protein kinase (*p38 MAPK*) in grass carp intestines.

### The mRNA levels of genes related to TJs in the intestines of grass carp

**Figure 6:**
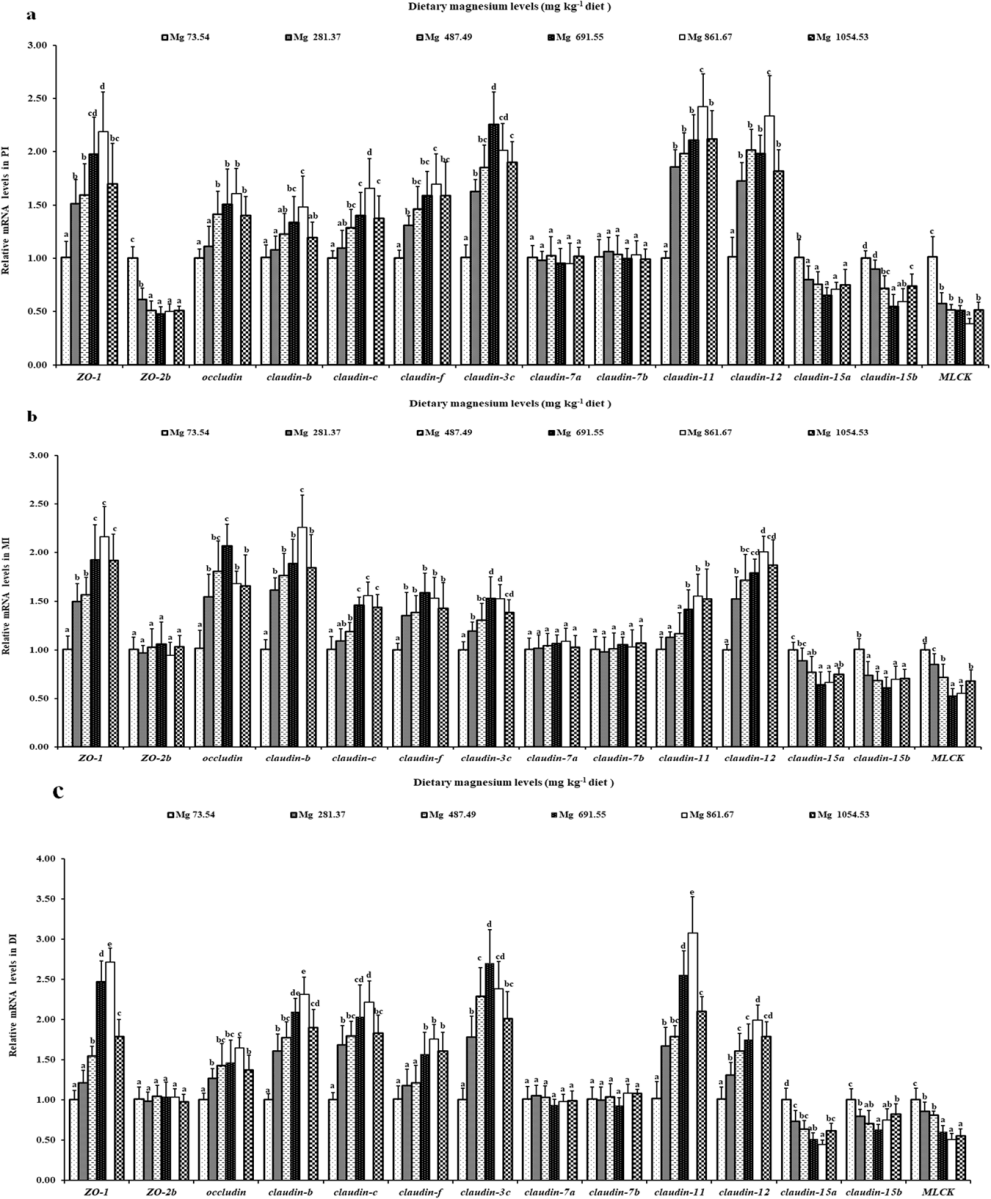
Relative gene expressions of tight junction complexes, transporter and *MLCK* in PI (**a**), MI (**b**) and DI (**c**) of grass carp fed diets containing graded levels of magnesium. Data represent means of six fish in each group, error bars indicate S.D. Values having different letters are significantly different (*P* < 0.05; ANOVA and Duncan’s multiple range tests).

Magnesium had multiple influences on TJs-related genes in grass carp intestines. In Fig. 6, it can be seen that the *claudin*-*b*, -*12*, -*11*, -*c* and *ZO*-*1* gene expression in grass carp intestines increased as the magnesium level rose to 861.67 mg/kg and decreased thereafter. Among all six groups, fish fed a magnesium-deficient diet showed the lowest levels of *claudin*-*3c* gene expression in grass carp intestines. Additionally, the gene expression of *occludin* and *claudin*-*f* was highest in grass carp DI and PI with a magnesium level of 861.67 mg/kg and in the MI with a magnesium level of 691.55 mg/kg and then decreased. However, fish in the magnesium-deficient group showed the highest levels of *claudin*-*15b* and *claudin*-*15a* gene expression in grass carp intestines. Meanwhile, *MLCK* gene expressions declined dramatically (*P* < 0.05) in grass carp DI and PI as the magnesium levels rose to 861.67 mg/kg and in the MI as.the level reached 691.55 mg/kg and then increased sharply (*P* < 0.05). Only in grass carp PI did the *ZO*-*2b* mRNA levels decrease significantly (*P* < 0.05) to a minimum at 281.37 mg/kg magnesium and then plateaued (*P* > 0.05). By accident, we found that dietary magnesium downregulated *ZO*-*2b* gene expression only in grass carp PI (rather than in the DI and MI) and that dietary magnesium had no influence on *claudin*-*7b* and -*7a* gene expression in the intestines of this fish.

## Discussion

In this study, we observed that magnesium deficiency suppressed grass carp (223.85–757.33 g) growth with poor FI, PWG, SGR and FE. It has been accepted that fish growth is related to nutrient metabolism in the fish body^41^. The magnesium nutritional level in the fish body can be reflected by the magnesium concentration in serum and tissues^41^. Our results showed that magnesium deficiency decreased the magnesium concentrations in grass carp serum and intestines.

Additionally, fish growth depends on the intestinal growth^45^. To our knowledge, fish intestinal growth can be reflected by the IL, ILI, IW and ISI^6^. According to our present data, magnesium deficiency depressed the IL, ILI, IW and ISI, suggesting that magnesium deficiency could depress fish intestinal growth. It has been generally accepted that fish intestinal growth depends on the intestinal structure^46^. One study demonstrated that goblet cell hyperplasia could thicken the mucus layer in the human intestine^47^. An excessively thickened mucus layer would block the intestinal absorption function in mammals^48,49^. Our histological results showed that magnesium deficiency caused goblet cell hyperplasia in grass carp intestines, which may partly contribute to the decreased activities of intestinal brush border enzymes (such as Na^+^, K^+^-ATPase and AKP). It has been demonstrated that AKP and Na^+^, K^+^-ATPase are involved in the absorption of nutrients (such as glucose and amino acids) in animal intestine^50,51^. In animal intestines, goblet cells are associated with the absorption of nutrients (such as glucose)^52^. In the present study, magnesium deficiency suppressed the Na^+^, K^+^-ATPase and AKP activities in fish intestines. We hypothesize that magnesium deficiency might decrease the activities of intestinal brush border enzymes (such as Na^+^, K^+^-ATPase and AKP), resulting in goblet cell hyperplasia to maintain the intestinal function of absorbing nutrients, an idea that needs more investigation. Magnesium deficiency-induced suppression of the Na^+^, K^+^-ATPase and AKP activities might be related to the physiological functions of magnesium. As is known, magnesium is involved in the active site of AKP in *Escherichia coli*^53^ and of Na^+^, K^+^-ATPase in animal kidney cells^54^. These results indicate that the depressed fish growth under a magnesium-deficient diet may be attributed to the suppression of intestinal brush border enzymes and the negative intestinal growth.

Undeniably, fish growth is related to the intestinal structural integrity, which depends on cellular and intercellular structural integrity^6^. Hence, it is imperative to study the relationship between magnesium deficiency and the cellular and intercellular structural integrity in fish intestines.

It was previously reported that ROS could induce oxidative damage and that ROS can be eliminated by the antioxidant system in fish^6^. Based on the current results, magnesium deficiency increased the contents MDA, PC and ROS while decreasing the antioxidant enzymes (except CuZnSOD) activities and the non-enzymatic antioxidant (GSH) content in grass carp intestines, indicating that magnesium deficiency increases oxidative damage because of decreasing antioxidant ability in fish intestines. To some extent, the gene expression of antioxidant-related enzymes can reflect the antioxidant enzyme activities in animals^55^. As the results show, magnesium deficiency decreased the antioxidant enzyme mRNA levels (except *CuZnSOD*) in grass carp intestines. Moreover, our study revealed that the antioxidant enzyme activities (GPx, GST, GR, CAT and MnSOD) had a positive correlation to the enzyme gene expression (Table 3). This evidence suggested that magnesium deficiency may downregulate antioxidant enzymes (except *CuZnSOD*) gene expression to decrease their activities in fish intestines.

**Table 3:** Correlation coefficients of genes relative expression in the intestine.

Independent parameters	Dependent parameters	PI	*P*	MI	*P*	DI	*P*
		Correlation coefficients		Correlation coefficients		Correlation coefficients	
*MnSOD* mRNA level	MnSOD activity	0.921	<0.01	0.935	<0.01	0.923	<0.01
*CAT* mRNA level	CAT activity	0.794	=0.060	—	—	—	—
*GPx1a* mRNA level	GPx activity	0.967	<0.01	0.911	<0.05	0.901	<0.05
*GPx1b* mRNA level		0.931	<0.01	0.967	<0.01	0.979	<0.01
*GPx4a* mRNA level		0.801	=0.056	0.965	<0.01	0.959	<0.01
*GPx4b* mRNA level		0.911	<0.05	0.963	<0.01	0.963	<0.01
*GSTR* mRNA level	GST activity	0.875	<0.05	0.891	<0.05	0.943	<0.01
*GSTO1* mRNA level		0.940	<0.01	0.987	<0.01	0.979	<0.01
*GSTO2* mRNA level		0.942	<0.01	0.860	<0.05	0.857	<0.05
*GSTP1* mRNA level		—	—	0.928	<0.01	0.963	<0.01
*GSTP2* mRNA level		0.904	<0.05	0.969	<0.01	0.983	<0.01
*GR* mRNA level	GR activity	0.847	<0.05	0.983	<0.01	0.925	<0.01
Nuclear Nrf2 protein level	*MnSOD*	0.916	=0.010	0.885	<0.05	—	—
	*CAT*	0.766	=0.076	0.938	<0.01	0.815	<0.05
	*GPx1a*	0.888	<0.05	0.942	<0.01	0.915	=0.010
	*GPx1b*	0.835	<0.05	0.925	<0.01	0.824	<0.05
	*GPx4a*	—	—	0.936	<0.01	0.871	<0.05
	*GP*x4b	0.820	<0.05	0.907	<0.05	0.920	<0.01
	*GSTR*	0.836	<0.05	0.898	<0.05	0.890	<0.05
	*GSTO1*	0.922	<0.01	0.966	<0.01	0.940	<0.01
	*GSTO2*	0.893	<0.05	0.853	<0.05	—	—
	*GSTP1*	—	—	0.979	<0.01	0.955	<0.01
	*GSTP2*	0.943	<0.01	0.894	<0.05	0.952	<0.01
	*GR*	0.818	<0.05	0.944	<0.01	0.934	<0.01
	*Keap1a*	−0.853	<0.05	−0.842	<0.05	−0.857	<0.05
*FasL*	*caspase*-*8*	0.950	<0.01	0.939	<0.01	0.963	<0.01
*caspase*-8	*caspase*-*3*	0.955	<0.01	0.963	<0.01	0.891	<0.05
	*caspase*-*7*	0.966	<0.01	0.938	<0.01	0.907	<0.01
*Bax*	*caspase*-*2*	0.938	<0.01	0.745	=0.089	0.955	<0.01
	*caspase*-*3*	0.882	<0.05	0.974	<0.01	0.859	<0.05
	*caspase*-*7*	0.921	<0.01	0.738	=0.094	0.950	<0.01
	*caspase*-*9*	0.955	<0.01	0.981	<0.01	0.945	<0.01
*Apaf*-1	*caspase*-*2*	0.944	<0.01	—	—	0.988	<0.01
	*caspase*-*3*	0.914	<0.05	0.962	<0.01	0.958	<0.01
	*caspase*-*7*	0.962	<0.01	0.767	=0.075	0.989	<0.01
	*caspase*-*9*	0.997	<0.01	0.888	<0.05	0.948	<0.01
*Bcl*-2	*caspase*-*2*	−0.868	<0.05	−0.829	<0.05	−0.944	<0.01
	*caspase*-*3*	−0.991	<0.01	−0.982	<0.01	−0.905	<0.05
	*caspase*-*7*	−0.989	<0.01	−0.903	<0.05	−0.972	<0.01
	*caspase*-*9*	−0.921	<0.01	−0.953	<0.01	−0.963	<0.01
*Mcl*-*1b*	*caspase*-*2*	−0.899	<0.05	—	—	−0.984	<0.01
	*caspase*-*3*	−0.852	<0.05	−0.917	<0.05	−0.937	<0.01
	*caspase*-*7*	−0.820	<0.05	−0.799	=0.057	−0.976	<0.01
	*caspase*-*9*	−0.765	=0.076	−0.810	=0.051	−0.956	<0.01
*IAP*	*caspase*-*2*	−0.923	<0.01	−0.878	<0.05	−0.953	<0.01
	*caspase*-3	−0.984	<0.01	−0.961	<0.01	−0.970	<0.01
	*caspase*-7	−0.958	<0.01	−0.933	<0.01	−0.970	<0.01
	*caspase*-*9*	−0.892	<0.05	−0.957	<0.01	−0.995	<0.01
*caspase*-*2*	*caspase*-*3*	0.924	<0.01	0.931	<0.01	0.893	<0.05
	*caspase*-*7*	0.940	<0.01	0.943	<0.01	0.936	<0.01
*caspase*-*9*	*caspase*-*3*	0.944	<0.01	0.960	<0.01	0.946	<0.01
	*caspase*-*7*	0.955	<0.01	0.899	<0.05	0.930	<0.01
*JNK*	*FasL*	0.983	<0.01	0.940	<0.01	0.898	<0.05
	*Apaf*-1	0.981	<0.01	0.926	<0.01	0.926	<0.01
	*Bax*	0.987	<0.01	0.977	<0.01	0.918	<0.05
	*IAP*	−0.927	<0.01	−0.975	<0.01	−0.993	<0.01
	*Bcl*-2	−0.920	<0.01	−0.977	<0.01	−0.963	<0.01
	*Mcl*-*1b*	−0.848	<0.05	−0.865	<0.05	−0.933	<0.01
*MLCK*	*occludin*	−0.853	<0.05	−0.879	<0.05	−0.863	<0.05
	*ZO*-*1*	−0.910	<0.05	−0.938	<0.01	−0.897	<0.05
	*ZO*-*2b*	0.971	<0.01	—	—	—	—
	*claudin*-*c*	−0.824	<0.05	−0.930	<0.01	−0.887	<0.05
	*claudin*-*f*	−0.946	<0.01	−0.952	<0.01	−0.992	<0.01
	*claudin*-*b*	−0.796	=0.058	−0.911	<0.05	−0.931	<0.01
	*claudin*-*3c*	−0.911	<0.05	−0.997	<0.01	−0.784	=0.065
	*claudin*-*11*	−0.991	<0.01	−0.862	<0.05	−0.927	<0.01
	*claudin*-*12*	−0.976	<0.01	−0.910	<0.05	−0.962	<0.01
	*claudin*-*15a*	0.932	<0.01	0.997	<0.05	0.913	<0.05
	*claudin*-*15b*	0.819	<0.05	0.875	<0.01	—	—

Interestingly, we found that dietary magnesium only enhanced *MnSOD* (not *CuZnSOD*) activity and gene expression in grass carp intestines, which may partly be attributed to apolipoprotein A-I (ApoA-I). It was reported that magnesium increased the concentration of ApoA-I in rats livers^56^ which only upregulated gene expression of *MnSOD* (not *CuZnSOD*) and incaresed MnSOD (not CuZnSOD) protein levels in mouse ID8 cells^57^, supporting our hypothesis.

Antioxidant enzyme gene expression is under Nrf2 signalling pathway regulation in mammals^58^. Nrf2 nuclear translocation could activate the Nrf2 signalling pathway, which could be evaluated by the nuclear Nrf2 protein level in mice^59^. Our results suggested that magnesium deficiency could decrease nuclear Nrf2 protein levels to suppress the nuclear translocation of Nrf2, which may inhibit the Nrf2 signalling pathway in fish intestines. Furthermore, our study showed that these antioxidant enzymes (except *CuZnSOD*) gene expressions had a positive connection to nuclear Nrf2 protein levels in grass carp intestines (Table 3), suggesting that magnesium deficiency may downregulate antioxidant enzyme gene expression by inhibiting the Nrf2 signalling pathway. The reasons for magnesium deficiency inhibiting Nrf2 nuclear translocation in fish intestines are as follows. First, the inhibition may be attributed to the *de novo* synthesis inhibition of Nrf2 by magnesium deficiency. It was reported that *de novo* synthesis inhibition of Nrf2 may block the Nrf2 nuclear translocation process in humans^60^. A previous study in mice reported that Nrf2 nuclear translocation was closely correlated with its transcriptional and translation levels^59^. Our study found that magnesium deficiency suppressed the total Nrf2 translational level (nuclear and cytosolic Nrf2 protein levels) and suppressed the *Nrf2* gene transcriptional level (*Nrf2* gene expression) in grass carp intestines, indicating that magnesium deficiency inhibited Nrf2 *de novo* synthesis to suppress Nrf2 nuclear translocation in fish intestines. The downregulation of *Nrf2* gene expression by magnesium deficiency in fish intestines may be attributed to vitamin B_6_ content. In rat plasma, magnesium deficiency could decrease the vitamin B_6_ content^61^. Our laboratory previous study observed that vitamin B_6_ deficiency decreased the *Nrf2* gene expression in grass carp intestines^62^. Therefore, magnesium deficiency might decrease the vitamin B_6_ content to downregulate the *Nrf2* gene expression in fish intestines. Second, the inhibition might be attributed to the upregulation of *keap1* gene expression by magnesium deficiency. In mice, Keap1 is a Nrf2-binding protein that prevents the Nrf2 nucleus translocation process by facilitating Nrf2 degradation^63^. The current study found that magnesium deficiency upregulated the *Keap1a* (not *Keap1b*) gene expression in grass carp intestines. Our study observed that *Keap1a* (not *Keap1b*) gene expression had a negative connection to nuclear Nrf2 protein levels in grass carp intestines (Table 3), indicating that the magnesium deficiency-induced suppression of Nrf2 nuclear translocation may occur partially via up-regulation of *Keap1a* (not *Keap1b*) gene expression in fish intestines. Our results above suggested that magnesium deficiency weakened the antioxidant capacity in fish intestines, which was partly associated with the *Nrf2*/*Keap1a* (not *Keap1b*) signalling pathway.

In contrast, we found that dietary magnesium downregulated only *Keap1a* (not *Keap1b*) gene expression in grass carp intestines, which may be associated with phospholipids. Gimenez *et al*.^64^ reported that magnesium could increase the phospholipids content in rat blood. Previously, our laboratory found that phospholipids decreased only *keap1a* (not *keap1b*) gene expression in juvenile grass carp intestines^7^, supporting our hypothesis.

Over all, we observed that magnesium deficiency decreased the antioxidant capacity and caused oxidative damage in fish intestines, which may be modulated by the *Nrf2*/*Keap1a* (not *Keap1b*) signalling pathway. A previous study found that oxidative damage aggravates cell apoptosis in humans^65^. Hence, we next examined the relationship between magnesium deficiency and cell apoptosis in fish intestines.

It is generally accepted that DNA fragmentation is a characteristic feature in cell apoptosis in humans^66^. Our research showed that magnesium deficiency could result in serious apoptosis in fish intestines. In addition, cell apoptosis-related gene expression in rats may partly reflect the degree of cell apoptosis^67^. Cell apoptosis is associated with apoptosis-related proteins [apoptosis activators (such as caspase-2, -8 and -9) and apoptosis executioners (such as caspase-3 and -7)] in mammals^68^. Previous studies demonstrated that proapoptotic proteins (Apaf-1and Bax) activated caspase-3, -2, -9 and -7 and that antiapoptotic proteins (*Mcl-1b, Bcl-2 and IAP*) inhibited caspase-3, -2, -9 and -7, while the proapoptotic protein FasL activated caspase-8 in humans^8^. In our present study, magnesium deficiency enhanced the gene expression of *caspase*-*3*, -*2*, -*8*, -*9*, -*7* and proapoptotic proteins (*Bax*, *FasL and Apaf*-*1*) but decreased that of antiapoptotic proteins (*Mcl*-*1b*, *Bcl*-2 *and IAP*) in grass carp intestines. Our study observed that *caspase*-*3*, -*2*, -*9 and* -*7* gene expression had a positive connection to proapoptotic protein (*Bax* and *Apaf*-*1*) gene expression, *caspase*-*3 and* -*7* gene expression had a positive connection to *caspase*-*2*, -*8 and* -*9* gene expression, and *caspase*-*8* gene expression had a positive connection to proapoptotic protein *FasL* gene expression, but *caspase*-*3*, -*2*, -*9 and* -*7* gene expression had a negative connection to antiapoptotic protein (*Mcl*-*1b*, *Bcl*-*2 and IAP*) gene expression in grass carp intestines (Table 3). These results indicated that magnesium deficiency may aggravate apoptosis in fish intestines and was partly dependent on [*FasL*/*caspase*-*8*/(*caspase*-*3 and* -*7*)] and [(*Bax*, *Apaf*-*1*, *Bcl*-*2*, *Mcl*-*1b and IAP*)/(*caspase*-*2* and -*9*)]/(*caspase*-*3* and -*7*)] signalling pathways.

In addition, JNK and p38MAPK take part in manipulating cell apoptosis in humans^69,70^. By coincidence, magnesium deficiency upregulated *JNK* gene expression but did not alter *p38MAPK* mRNA levels in grass carp intestines. The upregulation of *JNK* gene expression by magnesium deficiency in fish intestines may be attributed to a potassium deficiency. According to one study in animals, magnesium deficiency could cause potassium deficiency^71^. Potassium deficiency could also elevate the JNK protein level in calves^72^. Therefore, magnesium deficiency might cause a potassium deficiency, upregulating *JNK* gene expression in fish intestines. Afterwards, our study found that proapoptotic protein (*Bax*, *FasL* and *Apaf*-*1*) gene expression had a positive connection to *JNK* gene expression, but antiapoptotic protein (*Mcl*-*1b*, *Bcl*-*2* and *IAP*) gene expression had a negative connection to *JNK* gene expression in grass carp intestines (Table 3). In summary, all evidence above indicates that magnesium deficiency may aggravate apoptosis in fish intestines, partly depending on the [*JNK* (not *p3*8*MAPK*)/*FasL*/*caspase*-*8*/(*caspase*-*3* and -*7*)] and [*JNK* (not *p38MAPK*)/(*Bax*, *Apaf*-*1*, *Bcl*-*2*, *Mcl*-*1b* and *IAP*)/(*caspase*-*2* and -*9*)]/(*caspase*-*3* and -*7*)] signalling pathways.

Surprisingly, our study observed that magnesium did not alter *p38MAPK* gene expression in grass carp intestines, which may be attributed to vitamin D. According to a study of human blood, magnesium could increase the vitamin D content of blood^73^. Our previous research found that vitamin D did not alter *p38MAPK* gene expression in the enterocytes of fish^74^, supporting our hypothesis.

Moreover, TJs are always on the top of the list for maintaining intercellular structural integrity in human Caco-2 cells^75^, which is important for animal intestinal structural integrity^76^. Thus, an investigation of the relationship between magnesium deficiency and TJs in grass carp intestines as well as underlying signalling pathways is necessary.

TJs (such as occludin, claudins and ZO-1) could regulate the intercellular structural integrity in the sea bream (*Sparus aurata*) gut^77^. Research in mouse intestinal epithelia demonstrated that claudin-15 is one of the pore-forming proteins that improve epithelial permeability^78^. Based on our results, magnesium deficiency decreased *occludin*, *ZO*-*1*, and *claudin*-*c*, -*b*, -*3c*, -*f*, -*11* and -*12* gene expression, but upregulated *ZO*-*2b* (only in PI) and *claudin*-*15b* and -*15a* gene expression in grass carp intestines. One study reported that MLCK could regulate the tight junction permeability in terrestrial animals^79,80^, and the activation of MLCK could decrease TJ gene expression in the bovine brain^34^. Our data indicated that magnesium deficiency enhanced *MLCK* gene expression in grass carp intestines. The *MLCK* gene expression enhancement by magnesium deficiency in fish intestines may be attributed to an elevated concentration of TNF-α. It has been reported that magnesium deficiency elevated the concentration of TNF-α in humans^35^. Elevated TNF-α could also upregulate *MLCK* gene expression in humans^81^. Therefore, magnesium deficiency might elevate the TNF-α concentration to upregulate *MLCK* gene expression in fish intestines. Our study demonstrated that *occludin*, *ZO*-*1*, and *claudin*-*c*, -*b*, -*3c*, -*f*, -*11* and -*12* gene expression had a negative connection to *MLCK* gene expression, while *claudin*-1*5a* and -*15b* and *ZO*-*2b* (only in PI) gene expression had a positive connection to *MLCK* gene expression in grass carp intestines (Table 3). All evidence above suggests that magnesium deficiency damaged the tight junction function in fish intestines, which occurred partly through *MLCK* signalling pathway suppression of *occludin*, *ZO*-*1*, and *claudin*-*3c*, -*11*, -*b*, -*f*, -*c* and -*12* gene expression, and upregulation of *claudin*-*15a* and -*15b* and *ZO*-*2b* (only in PI) gene expression.

Surprisingly, we found that dietary magnesium deficiency increased *ZO*-*2b* gene expression only in grass carp PI (rather than DI and MI) and that dietary magnesium had no influence on *claudin*-*7a* and -*7b* gene expression in the intestines of this fish. Several reasonable potential causes for these effects are as follows. First, dietary magnesium deficiency upregulated the *ZO*-*2b* gene expression only in grass carp PI (rather than DI and MI), which may be attributed to the zinc in fish intestines. It was reported that magnesium deficiency increased the intestinal absorption of zinc in rats^82^. Our laboratory previous study observed that zinc increased the *ZO*-*2b* gene expression only in grass carp PI (rather than DI and MI)^83^, supporting our hypothesis. Second, dietary magnesium did not alter *claudin*-*7a* and -*7b* gene expression in grass carp intestines, which may be attributed to Na^+^, K^+^-ATPase in fish intestines. Previously, Alexandre *et al*.^84^ reported that claudin-7 is generally accepted as a channel for Na^+^ in pig LLC-PK1 cells. Moreover, magnesium could activate human blood Na^+^, K^+^-ATPase activity^85^, which regulates Na^+^ movement in most higher eukaryotes^86^. Hence, we suggest that dietary magnesium may enhance the Na^+^, K^+^-ATPase activity to regulate Na^+^ movement instead of claudin-7, resulting in the observed stable gene expression of *claudin*-*7b* and -*7a* in fish intestines. However, this hypothesis deserves deeper research.

Meaningfully, in this study, there are some innovative discoveries of magnesium beyond the previous knowledge of magnesium. We list these novel discoveries as follows: (1) Previous researches involving the effect of magnesium on oxidative damage in aminals has only focused on the oxidation products (ROS, MDA and PC) and antioxidant enzymes (SOD, GST, GPX and CAT)^87–91^. However, apart from the investigation of oxidation products (ROS, MDA and PC), antioxidant enzymes (SOD, GR, GST, GPX and CAT) and non-enzymatic antioxidants (GSH), we studied the mRNA levels of genes corresponding to antioxidant enzymes (*CuZnSOD*, *MnSOD*, *CAT*, *GR*, *GPx1a*, *GPx1b*, *GPx4a*, *GPx4b*, *GSTR*, *GSTP1*, *GSTP2*, *GSTO1* and *GSTO2*) and evaluated the Nrf2 nuclear translocation level associated with nuclear Nrf2 protein levels and cytosolic Nrf2 protein levels as well as the mRNA levels of signalling molecules (*Keap1a*, *Keap1b* and *Nrf2*) in fish intestines. It has been generally accepted that Nrf2 nuclear translocation could activate the Nrf2 signalling pathway to regulate the genes expressions of antioxidant enzymes in mammals^58^. Our results innovatively found that magnesium deficiency may suppress the nuclear translocation of Nrf2 to inhibit the Nrf2 signalling pathway and downregulate the expression of genes corresponding to antioxidant enzymes, which then decreases the antioxidant enzyme activities in fish intestines. (2) The caspase family proteins (such as caspase-2, -3, -7, -8 and -9) are mainly in charge of cell apoptosis in organisms^8^. In the caspase family, apoptosis executioner (such as caspase-3 and -7) can directly regulate cell apoptosis in organisms^8^. Apart from the apoptosis executioners (such as caspase-3 and -7), the apoptosis activators (such as caspase-2, -8 and -9) of the caspase family are the upstream signalling molecules of apoptosis executioners (such as caspase-3 and -7), which can directly activate the apoptosis executioners (such as caspase-3 and -7) to regulate cell apoptosis in organisms^8^. Additionally, there are two distinctly-different apoptotic pathways (the death receptor pathway and the mitochondria pathway) in organisms, and the pathways include several signalling molecules [pro-apoptotic proteins (FasL, Apaf-1 and Bax) as well as anti-apoptotic proteins (Bcl-2, Mcl-1b and IAP)] to regulate the caspases (caspase-2, -3, -7, -8 and -9) taking part in cell apoptosis^92^. However, previous researches involving the effect of magnesium on cell apoptosis in animals has only focused on one apoptosis executioner (caspase-3 activity), a protein which is part of the terminal apoptosis signalling pathway^11,93^. Surprisingly, in our present study, we innovatively found that magnesium deficiency could upreguate the gene expressions of *caspase*-*2*, -*3*, -*7*, -*8*, -*9* and pro-apoptotic proteins (*FasL*, *Apaf*-*1* and *Bax*), but downregulate the gene expressions of anti-apoptotic proteins (*Bcl*-*2*, *Mcl*-*1b* and *IAP*) to activate the death receptor pathway [*FasL*/*caspase*-*8*/(*caspase*-*3* and -*7*)] and the mitochondria pathway [(*Bax*, *Apaf*-*1*, *Bcl*-*2*, *Mcl*-*1b* and *IAP*)/(*caspase*-*2* and -*9*)]/(*caspase*-*3* and -*7*)], aggravating cell apoptosis in fish intestines. (3) It is generally accepted that scaffolding proteins (such as ZO-1 and ZO-2), barrier-forming proteins (such as claudin-c, -3, -f, -b, -11, -7 and occludin) and pore-forming TJs (such as claudin-12 and -15) of tight junctional complexes (TJs) play different roles in corporately maintaining the animal intercellular integrity^94–96^. Additionally, MLCK can induce contraction of the perijunctional actomyosin ring that encircles the cell at the adherens junction and TJ through myosin II regulatory light chain phosphorylation to regulate the tight junction permeability in terrestrial animals^79^. Until now, only one previous study involving the effect of magnesium on TJs in animals focused only on one of the scaffolding proteins (ZO-1) and one of the barrier-forming proteins (occludin)^5^. However, our present study observed that magnesium deficiency could downregulate the mRNA levels of scaffolding proteins (*ZO*-*1*), barrier-forming proteins (*claudin*-*c*, -*3c*, -*f*, -*b*, and -*11* and *occludin*) and pore-forming TJs (*claudin*-*12*), but upregulate the mRNA levels of scaffolding proteins *ZO*-*2b* (only in PI), pore-forming TJs (*claudin*-*15a* and -*15b*) and *MLCK*, indicating that magnesium deficiency could activate the MLCK signalling pathway to regulate the tight junctional complex (TJs) function in animals.

**Figure 7:**
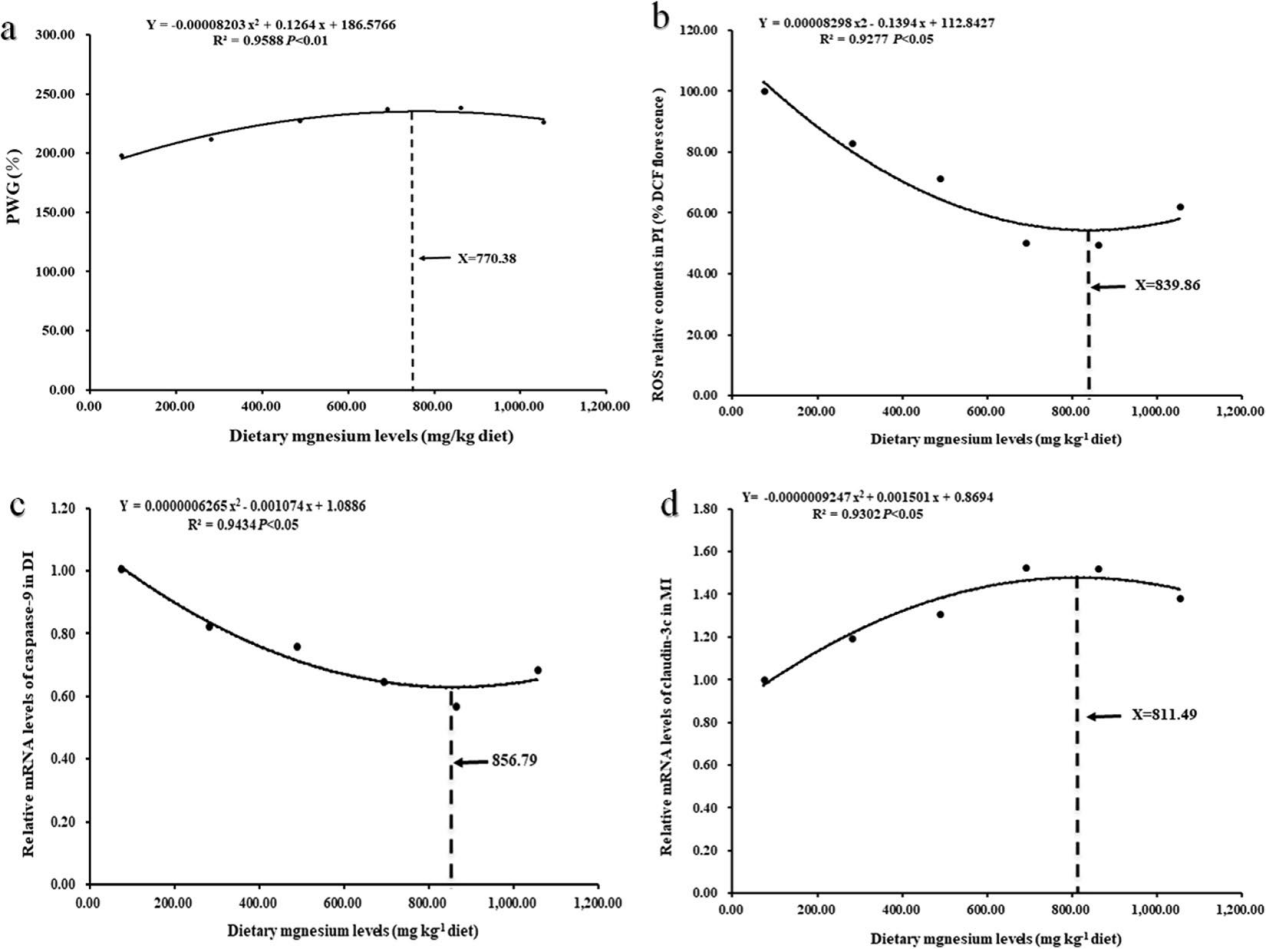
Quadratic regression analysis of PWG (%) (**a**), ROS (% DCF florescence) (**b**) and relative mRNA levels of *caspase*-9 (**c**) as well as *claudin*-*3c* (**d**) of grass carp fed diets containing graded levels of magnesium.

In the present study, we investigated the magnesium requirement of grass carp (223.85–757.33) g based on growth performance. Based on the PWG of grass carp (223.85–757.33 g), the optimal dietary magnesium level was calculated to be 770.38 mg/kg (Fig. 7), which is slightly higher than that of juvenile grass carp (7.69–35.90 g), for which 713.50 mg/kg was recommended by Wang *et al*.^41^. The reason for the different optimal dietary magnesium levels between grass carp (223.85–757.33 g) and juvenile grass carp (7.69–35.90 g) may be attributed to the different growth rates of juvenile grass carp (7.69–35.90 g) and grass carp (223.85–757.33 g). As is known, due to a higher growth rate, the nutrient requirements (such as available phosphorus) for juvenile fish are higher than those for young fish^25,97^. Interestingly, however, we found that the juvenile grass carp growth rate (2.01%/day) recommended by Wang *et al*.^41^ is much lower than the normal juvenile grass carp growth rate (3.07%/day) recommended by Dong *et al*.^98^. Additionally, the juvenile grass carp (7.69–35.90 g) growth rate (2.01%/day) recommended by Wang *et al*.^41^ is close to the grass carp (223.85–757.33 g) growth rate (2.03%/day) in our study, which resulted in the slightly higher magnesium requirement for grass carp (223.85–757.33 g).

Simultaneously, we also investigated the magnesium requirement of grass carp (223.85–757.33 g) based on intestinal structural integrity related indices. Based on against ROS, against *caspase*-*9* and *claudin*-*3c* in grass carp (223.85–757.33 g) (Fig. 7), the optimal magnesium levels were calculated to be 839.86, 856.79 and 811.49 mg/kg, respectively. Comparatively, the optimal magnesium levels based on intestinal structural integrity related indices were higher than those based on growth performance (PWG), indicating that more magnesium might be needed to maintain intestinal structural integrity in fish. This can be attributed to the additional requirements of antimicrobial-related enzymes (acid phosphatase and AKP) in fish intestines. As our data shows, pathogen invasion (such as *A*. *hydrophila*) could impair fish intestinal structural integrity. Additionally, it has been reported that magnesium could enhance the activities of AKP in calf intestines^99^ and of acid phosphatase in carp (*Cyprinus carpio* L.)^100^. AKP could detoxify lipopolysaccharides and prevent pathogens in Zebrafish (*Danio rerio*)^101^, and acid phosphatase can be a marker of the digestive capacity of phagocytes, which are associated with the elimination of pathogens in fish^102^. Therefore, we assumed that when pathogens invaded, fish would need more magnesium to enhance the antimicrobial-related enzyme activities and maintain intestinal structural integrity.

## Conclusion

**Figure 8:**
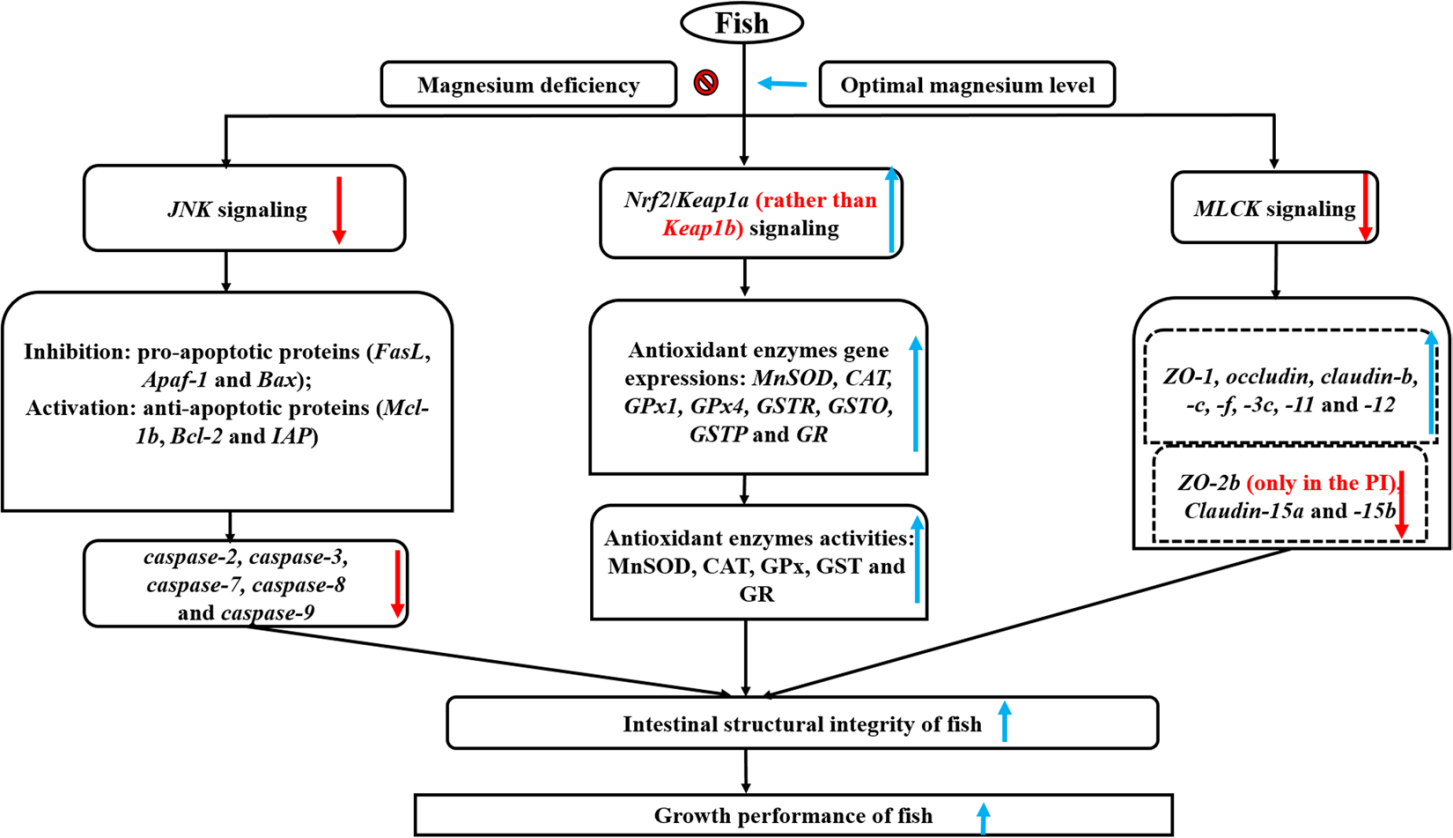
Potential action pathways of dietary magnesium regulate intestinal structural integrity in fish.

Looking back to our research (Fig. 8), magnesium deficiency suppressed fish growth, and we systematically investigated the impacts of magnesium deficiency on structural integrity in fish intestines. The following novel results were discovered in this study. (1) Magnesium deficiency weakened the antioxidant ability to impair the cellular structural integrity, which was attributed to the suppression of Nrf2 nuclear translocation that inhibited the Nrf2 signalling pathway to decrease antioxidant enzyme activities and gene expression (except *CuZnSOD* gene expressions and activities) in fish intestines. (2) Magnesium deficiency aggravated cell apoptosis to impair the cellular structural integrity through up-regulation of the *JNK* mRNA level (not *p38MAPK*), which increased *caspase*-*3*, -*2*, -*8*, -*7* and -*9* and proapoptotic protein (*Apaf*-*1*, *FasL* and *Bax*) gene expression but decreased antiapoptotic protein (*Mcl*-*1b*, *Bcl*-*2* and *IAP*) gene expression in fish intestines. (3) Magnesium deficiency damaged the TJ function to impair the intercellular structural integrity, which was associated with upregulated gene expression of *MLCK*, which decreased the relevant TJ gene expression (except *claudin*-*15b*, -*7b*, *ZO*-*2b*, *claudin*-*15a* and *7a* gene expression) in fish intestines. Additionally, based on PWG, against ROS, against *caspase*-*9* and *claudin*-*3c* in grass carp (223.85–757.33 g), the optimal dietary magnesium levels were calculated to be 770.38, 839.86, 856.79 and 811.49 mg/kg, respectively.

## Materials and Methods

This study was approved by the Institutional Animal Care and Use Committee of the Sichuan Agricultural University, Sichuan, China under permit No.DKY-S20150812. All experimental procedures concerning animals were in accordance with the Animal Management Rules of the Ministry of Health of the People’s Republic of China (Documentation 55, 2001, Ministry of Health, China).

### Experimental designs for diets

Feed contents and nutrient levels are presented in Table 4. Magnesium sulfate (MgSO_4_·H_2_O) was supplemented at 0 (control diet), 200, 400, 600, 800 and 1000 mg/kg in the basal diet. According to atomic absorption spectrometry^103^, the dietary magnesium actual concentrations were measured to be 73.54 (control diet), 281.37, 487.49, 691.55, 861.67 and 1054.53 mg/kg, respectively. The diets preparation measures and the storage methods were according to Wang *et al*.^41^.

**Table 4:** Composition and nutrients content of basal diet.

Ingredients	g kg^−1^	Nutrient contents^||^	g kg^−1^
Casein	240.00	Crude protein	288.31
Gelatin	93.60	Crude lipid	53.87
α-starch	240.00	ω-3	10.40
Corn starch	258.80	ω-6	9.60
Fish oil	29.30	Available phosphorus	4.00
Soy bean oil	18.00		
Magnesium premix^†^	10.00		
Vitamin premix^‡^	10.00		
Mineral premix^§^	20.00		
Ca (H_2_PO_4_)_2_	16.00		
L-Trp (99%)	0.900		
DL-Met (99%)	2.900		
Choline chloride (60%)	10.00		
Cellulose	50.00		
Ethoxyquin (30%)	0.50		

^†^Magnesium premix: premix was added to obtain graded concentrations of magnesium.

^‡^Per kilogram of vitamin premix (g kg^−1^): retinyl acetate (500,000 IU g^−1^), 0.39; cholecalciferol (500,000 IU g^−1^), 0.40; D, L-α-tocopherol acetate (50%), 23.23; menadione (22.9%), 0.83; cyanocobalamin (1%), 0.94; D-biotin (2%), 0.75; folic acid (95%), 0.42; thiamine nitrate (98%), 0.09; ascorhyl acetate (95%), 9.77; niacin (99%), 4.04; meso-inositol (98%), 19.39; calcium-D-pantothenate (98%), 3.85; riboflavin (80%), 0.73; pyridoxine hydrochloride (98%), 0.62. All ingredients were diluted with corn starch to 1 kg.

^§^Per kilogram of mineral premix (g kg^−1^): MnSO_4_.H_2_O (31.8% Mn), 2.6590; FeSO_4_.H_2_O (30.0% Fe), 12.2500; ZnSO_4_.H_2_O (34.5% Zn), 8.2460; CuSO_4_.5H_2_O (25.0% Cu), 0.9560; KI (76.9% I), 0.0650; Na_2_SeO3 (44.7% Se), 0.0168. All ingredients were diluted with corn starch to 1 kg.

^||^Crude protein and Crude lipid contents were measured value. Crude protein content was referenced to Xu *et al*.^32^, Available phosphorus content was referenced to Wen *et al*.^116^ and calculated according to NRC (2011), ω-3 and ω-6 contents were referenced to Zeng *et al*.^117^ and calculated according to NRC (2011).

### Experimental process and sample collection

Fish in this study were got from the fisheries of Sichuan, China. Prior to the growth trial, the grass carp were supplied with the control diet for 14 days according to Wang *et al*.^41^. After a 14 days acclimatization period, the total 540 grass carp [approximately 223.85 (SD 0.49) g] were randomly allocated into six treatments and each treatment has triplicate cages (30 fish/cage in each cage). And each cage (1.4 L × 1.4 W × 1.4 H m) was furnished with a round plate (diameter 100.00 cm) at the bottom, aiming to collect the residual feed. During growth trial, grass carp were supplied with their corresponding diet to apparent satiety four times per day. Any uneaten feed were collected thirty minutes later after feeding, then the feed not eaten were dried and weighed to calculate the feed intake (FI). In the experiment period, water magnesium concentration was determined to be 7.42 (SD 2.58) mg/L. The pH and water temperature were determined to be 7.5 (SD 0.3) and 28.5 (SD 2) °C, respectively. Also, oxygen content in water was maintained above 6.0 mg/L. Before and after growth trial, all grass carp in each treatment were weighed for calculating the growth performance related parameters. Later, all fish were anaesthetized with benzocaine before sacrificed. Then, the fish intestines were quickly separated and segmented into proximal intestine (PI), mid intestine (MI) and distal intestine (DI). After that, fish intestines were weighed and measured to calculate the intestinal related indices, and to measure the magnesium concentrations as well as the AKP and Na^+^, K^+^-ATPase activities. The grass carp blood samples in six treatments were obtained from fish bodies. Subsequently, the blood samples of grass carp were separated to get the serum which was saved in the −80 °C for measuring serum magnesium concentration.

After the growth trial, using the prevalent pathogens to impair the fish intestinal structural integrity is a common approach to evaluate the nutritional protection on fish intestinal structural integrity^104^. The *A*. *hydrophila* is a popular pathogen which could impair fish intestinal structural integrity^83^. After a 60 days growth trial, fifteen grass carp in similar body weight were selected from each treatment group to inject the *A*. *hydrophila* into fish bodies intraperitoneally for 14 days challenge trial (Fig. S1). Besides, another fifteen grass carp were selected from the control diet group to inject physiological saline into fish bodies intraperitoneally. After the challenge trial, fish were anaesthetized with benzocaine before sacrificed. Subsequently, all fish intestines were quickly separated and segmented into three segments. And the samplings were saved in the −80 °C until analysis.

### Sample preparation and biochemical parameters analysis

The intestines samples were homogenized in 10 volumes (w/v) of ice-cold physiological saline to get the homogenate. After that, the homogenate was centrifuged at 6000 *g* for 20 min at 4 °C to collect the supernatant which was saved for subsequent analysis of related parameters. The malondialdehyde (MDA), ROS, glutathione (GSH) and protein carbonyl (PC) contents were determined according to previous studies^105,106^. The anti-hydroxy radical (AHR) and anti-superoxide anion (ASA) capacities were determined according to Feng *et al*.^107^. Besides, the copper, zinc superoxide dismutase (CuZnSOD), total superoxide dismutase (SOD), catalase (CAT), glutathione-S-transferases (GST) and glutathione peroxidase (GPx) activities were determined as described by pervious studies^108,109^. The activity of glutathione reductase (GR) was measured according to Yang *et al*.^110^. Additionally, the total SOD activity minus CuZnSOD activity to get the manganese superoxide dismutase (MnSOD) activity. The analytical methods of the magnesium concentration in serum and in grass carp intestines are similar to Wang *et al*.^41^. The intestinal alkaline phosphatase (AKP) and NA^+^-K^+^-ATPase activities can be measured according to previous study^111^.

### Histological changes

Intestinal histological samples were rinsed in saline and preserved in 4% paraformaldehyde solution. Subsequently, the preserved intestinal samples were clear and dehydrated in a series of increasing ethanol concentrations (70%, 80%, 85%, 90%, 95% and 100%). After that, the tissues were prepared for being embedded in paraffin wax and sectioned to 4 mm. And sections were prepared for using standard hematoxylin and eosin (H & E) to be stained as described by Wang *et al*.^112^. After stained, the histological sections were examined by using a Nikon TS100 light microscope.

### Detection of fragmentation in DNA

The DNA fragmentation in different intestinal segments was isolated with reference to Kawakami *et al*.^113^. Fragmented DNA was assayed by agarose gel electrophoresis. The DNA was loaded on to the 2.0% agarose gel, and then electrophoresis was carried out at 80 V for 1.5 h. The gel was visualized and photographed by the Gene Genius Bio-Imaging system (Syngene, Frederick, MD, USA).

### Analysis of real-time PCR

The total RNA of fish intestines was isolated with RNAiso Plus kit (TaKaRa, Dalian, Liaoning, China). Single-stranded cDNA was prepared from total RNA by using the PrimeScript™ RT reagent Kit (TaKaRa) to reverse transcription. PCR Specific primers were designed based on gene sequences that were publicly available at gene bank of grass carp and cloned by our laboratory for quantitative real-time PCR (Table 5). By evaluating the internal control genes in our preliminary experiment (Tables S1–S3), β-actin was used as a reference gene to normalize cDNA loading. According to the specific gene standard curves, the housekeeping and target gene amplification efficiency were calculated. Besides, the amplification efficiency of primers are listed in Table 5. According to Schmittgen *et al*.^114^, the calculation method in expression results was the 2^−ΔΔCT^ method.

**Table 5:** Real-time PCR primer sequences*.

Target gene	Primer sequence Forward (5′ → 3′)	Primer sequence Reverse (5′ → 3′)	Amplification efficiency (%)	Temperature (°C)	Accession number
*occludin*	TATCTGTATCACTACTGCGTCG	CATTCACCCAATCCTCCA	99.7	59.4	KF193855
*ZO*-*1*	CGGTGTCTTCGTAGTCGG	CAGTTGGTTTGGGTTTCAG	100.0	59.4	KJ000055
*ZO*-*2b*	TACAGCGGGACTCTAAAATGG	TCACACGGTCGTTCTCAAAG	99.7	60.3	KM112095
*claudin*-*b*	GAGGGAATCTGGATGAGC	ATGGCAATGATGGTGAGA	100.9	57.0	KF193860
*claudin*-*c*	GAGGGAATCTGGATGAGC	CTGTTATGAAAGCGGCAC	100.6	59.4	KF193859
*claudin*-*f*	GCTGGAGTTGCCTGTCTTATTC	ACCAATCTCCCTCTTTTGTGTC	99.0	57.1	KM112097
*claudin*-*3c*	ATCACTCGGGACTTCTA	CAGCAAACCCAATGTAG	99.9	57.0	KF193858
*claudin*-*7a*	ACTTACCAGGGACTGTGGATGT	CACTATCATCAAAGCACGGGT	100.2	59.3	KT625604
*claudin*-*7b*	CTAACTGTGGTGGTGATGAC	AACAATGCTACAAAGGGCTG	100.0	59.3	KT445866
*claudin*-*11*	TCTCAACTGCTCTGTATCACTGC	TTTCTGGTTCACTTCCGAGG	100.3	62.3	KT445867
*claudin*-*12*	CCCTGAAGTGCCCACAA	GCGTATGTCACGGGAGAA	99.8	55.4	KF998571
*claudin*-*15a*	TGCTTTATTTCTTGGCTTTC	CTCGTACAGGGTTGAGGTG	99.6	59.0	KF193857
*claudin*-*15b*	AGTGTTCTAAGATAGGAGGGGAG	AGCCCTTCTCCGATTTCAT	99.9	62.3	KT757304
*MLCK*	GAAGGTCAGGGCATCTCA	GGGTCGGGCTTATCTACT	100.5	53.0	KM279719
*FasL*	AGGAAATGCCCGCACAAATG	AACCGCTTTCATTGACCTGGAG	100.0	61.4	KT445873
*p38 MAPK*	TGGGAGCAGACCTCAACAAT	TACCATCGGGTGGCAACATA	99.7	60.4	KM112098
*JNK*	ACAGCGTAGATGTGGGTGATT	GCTCAAGGTTGTGGTCATACG	100.4	62.3	KT757312
*Bcl*-*2*	AGGAAAATGGAGGTTGGGAT	CTGAGCAAAAAAGGCGATG	100.0	60.3	JQ713862.1
*Mcl*-*1b*	TGGAAAGTCTCGTGGTAAAGCA	ATCGCTGAAGATTTCTGTTGCC	100.8	58.4	KT757307
*Bax*	CATCTATGAGCGGGTTCGTC	TTTATGGCTGGGGTCACACA	100.0	60.3	JQ793788.1
*Apaf*-*1*	AAGTTCTGGAGCCTGGACAC	AACTCAAGACCCCACAGCAC	100.1	61.4	KM279717
*IAP*	CACAATCCTGGTATGCGTCG	GGGTAATGCCTCTGGTGCTC	99.7	58.4	FJ593503.1
*caspase*-*2*	CGCTGTTGTGTGTTTACTGTCTCA	ACGCCATTATCCATCTCCTCTC	99.0	60.3	KT757313
*caspase*-*3*	GCTGTGCTTCATTTGTTTG	TCTGAGATGTTATGGCTGTC	100.0	55.9	JQ793789
*caspase*-*7*	GCCATTACAGGATTGTTTCACC	CCTTATCTGTGCCATTGCGT	100.0	57.1	KT625601
*caspase*-*8*	ATCTGGTTGAAATCCGTGAA	TCCATCTGATGCCCATACAC	100.0	59.0	KM016991
*caspase*-*9*	CTGTGGCGGAGGTGAGAA	GTGCTGGAGGACATGGGAAT	99.4	59.0	JQ793787
*Cu*-*Zn/SOD*	CGCACTTCAACCCTTACA	ACTTTCCTCATTGCCTCC	100.3	61.5	GU901214
*MnSOD*	ACGACCCAAGTCTCCCTA	ACCCTGTGGTTCTCCTCC	99.2	60.4	GU218534
*CAT*	GAAGTTCTACACCGATGAGG	CCAGAAATCCCAAACCAT	100.0	58.7	FJ560431
*GPx1a*	GGGCTGGTTATTCTGGGC	AGGCGATGTCATTCCTGTTC	100.0	61.5	EU828796
*GPx1b*	TTTTGTCCTTGAAGTATGTCCGTC	GGGTCGTTCATAAAGGGCATT	100.0	60.3	KT757315
*GPx4a*	TACGCTGAGAGAGGTTTACACAT	CTTTTCCATTGGGTTGTTCC	99.9	60.4	KU255598
*GPx4b*	CTGGAGAAATACAGGGGTTACG	CTCCTGCTTTCCGAACTGGT	100.1	60.3	KU255599
*GSTR*	TCTCAAGGAACCCGTCTG	CCAAGTATCCGTCCCACA	99.6	58.4	EU107283
*GSTP1*	ACAGTTGCCCAAGTTCCAG	CCTCACAGTCGTTTTTTCCA	100.0	59.3	KM112099
*GSTP2*	TGCCTTGAAGATTATGCTGG	GCTGGCTTTTATTTCACCCT	100.0	59.3	KP125490
*GSTO1*	GGTGCTCAATGCCAAGGGAA	CTCAAACGGGTCGGATGGAA	100.2	58.4	KT757314
*GSTO2*	CTGCTCCCATCAGACCCATTT	TCTCCCCTTTTCTTGCCCATA	99.9	61.4	KU245630
*GR*	GTGTCCAACTTCTCCTGTG	ACTCTGGGGTCCAAAACG	99.5	59.4	JX854448
*Nrf2*	CTGGACGAGGAGACTGGA	ATCTGTGGTAGGTGGAAC	100.6	62.5	KF733814
*Keap1a*	TTCCACGCCCTCCTCAA	TGTACCCTCCCGCTATG	100.2	63.0	KF811013
*Keap1b*	TCTGCTGTATGCGGTGGGC	CTCCTCCATTCATCTTTCTCG	99.0	57.9	KJ729125
*β*-*actin*	GGCTGTGCTGTCCCTGTA	GGGCATAACCCTCGTAGAT	100.0	61.4	M25013

### Western blotting

Protein homogenates were prepared from intestines. Antibodies incubation and western blot analysis were processed according to Jiang *et al*.^115^. When the intestinal protein was extracted, the concentrations of protein were measured by using corresponding assay kit. After being separated by SDS-PAGE, protein samples (40 μg per lane) were transferred to a PVDF membrane for analysis of western blot. After being blocked at room temperature for 1.5 h, then the membrane was incubated overnight at 4 °C with primary antibody. The nuclear Nrf2, cytosolic Nrf2, Lamin B1and β-Actin antibodies were the same with those in previous study^115^. In this study, nuclear Nrf2 employed Lamin B1 as control proteins and cytosolic Nrf2 used β-Actin for control proteins. Subsequently, the PVDF membrane got washed with TBST for three times with 5 minutes each time and got incubated with goat anti-rabbit horseradish peroxidase-conjugated secondary antibody (Santa Cruz Biotechnology, Santa Cruz, CA, USA) in TBST for 2 h. The immunoreactive bands were visualized by ECL reagents (Beyotime Biotechnology Inc., China). The density of protein bands were detected by using NIH Image 1.63 software. Results for all protein levels by densitometric analyses were expressed as the fold of nucleotides treatment groups relative to the control group. The western blotting result from each group were measured there times independently.

### Data statistics and analysis

Growth performance parameters were calculated, and the formulas were followed: growth performance was assessed based on SGR, PWG and FE, which were in term of the data of final body weight (FBW), initial body weight (IBW) and FI. The data of intestinal somatic index (ISI) and intestinal length index (ILI) were calculated based on the data of intestinal weight (IW) and intestinal length (IL) according to Li *et al*.^6^.

$$\begin{array}{rcl}{\rm{PWG}}\,( \% ) & = & 100\times [{\rm{FBW}}({\rm{g}}/{\rm{fish}})-{\rm{IBW}}({\rm{g}}/{\rm{fish}})]/{\rm{IBW}}({\rm{g}}/{\rm{fish}});\\ {\rm{SGR}}( \% /{\rm{day}}) & = & 100\times \,\mathrm{ln}\,[{\rm{FBW}}({\rm{g}})/{\rm{IBW}}({\rm{g}})]/{\rm{days}};\\ {\rm{FE}}\,( \% ) & = & 100\times [{\rm{FBW}}({\rm{g}}/{\rm{fish}})-{\rm{IBW}}({\rm{g}}/{\rm{fish}})]/{\rm{FI}}({\rm{g}}/{\rm{fish}});\\ {\rm{ILI}} & = & 100\times [{\rm{IL}}({\rm{cm}})/{\rm{total}}\,{\rm{body}}\,{\rm{length}}({\rm{cm}})];\\ {\rm{ISI}} & = & 100\times [{\rm{IW}}({\rm{g}})/{\rm{wet}}\,{\rm{body}}\,{\rm{weight}}({\rm{g}})];\end{array}$$

The analysis method of data was the one-way analysis of variance (ANOVA), and the significant differences between the treatments were calculated by Duncan’s multiple-range test with SPSS 19.0 (SPSS Inc., Chicago, IL, USA) at *P* < 0.05. Based on PWG and structural integrity related indicators, the optimal dietary magnesium levels were calculated by quadratic regression model as described by Wang *et al*.^41^.

## Electronic supplementary material

Supplementary Information
