# Supplementary material for: Dietary magnesium deficiency impaired intestinal structural integrity in grass carp (Ctenopharyngodon idella)
Source: Sci Rep. 2018 Aug 23;8:12705. doi: 10.1038/s41598-018-30485-8 (PMC6107577; doi:10.1038/s41598-018-30485-8)
Supplement: Supplementary file 1 — Supplementary Information [file 41598_2018_30485_MOESM1_ESM.doc]

**Dietary magnesium deficiency impaired intestinal structural integrity in grass carp** **(*Ctenopharyngodon idella*)**

Shuo-Peng Wei^1^†, Wei-Dan Jiang^1,2,3^†, Pei Wu^1,2,3^, Yang Liu^1,2,3^, Yun-Yun Zeng^1,2,3^, Jun Jiang^1^, Sheng-Yao Kuang^4^, Ling Tang^4^, Yong-An Zhang^5^, Xiao-Qiu Zhou^1,2,3^*, Lin Feng^1,2,3^*

^1^ Animal Nutrition Institute, Sichuan Agricultural University, Chengdu 611130, China

^2^ Fish Nutrition and safety Production University Key Laboratory of Sichuan Province, Sichuan Agricultural University, Chengdu 611130, China

^3^ Key Laboratory for Animal Disease-Resistance Nutrition of China Ministry of Education, Sichuan Agricultural University, Chengdu 611130, China

^4^ Animal Nutrition Institute, Sichuan Academy of Animal Science, Chengdu 610066, China

^5^ Institute of Hydrobiology, Chinese Academy of Sciences, Wuhan 430072, China

*Corresponding authors. Animal Nutrition Institute, Sichuan Agricultural University, Chengdu 611130, Sichuan, China. Tel.: +86 835 2885157; fax: + 86 8352 885968

E-mail addresses: xqzhouqq@tom.com, zhouxq@sicau.edu.cn (X.-Q. Zhou); fenglin@sicau.edu.cn (L. Feng).

†These two authors contribute to this work equally

**Supplementary Materials:**

Supplementary figure. S1 to S2

Supplementary table. S1 to S3

**Figure. S1**


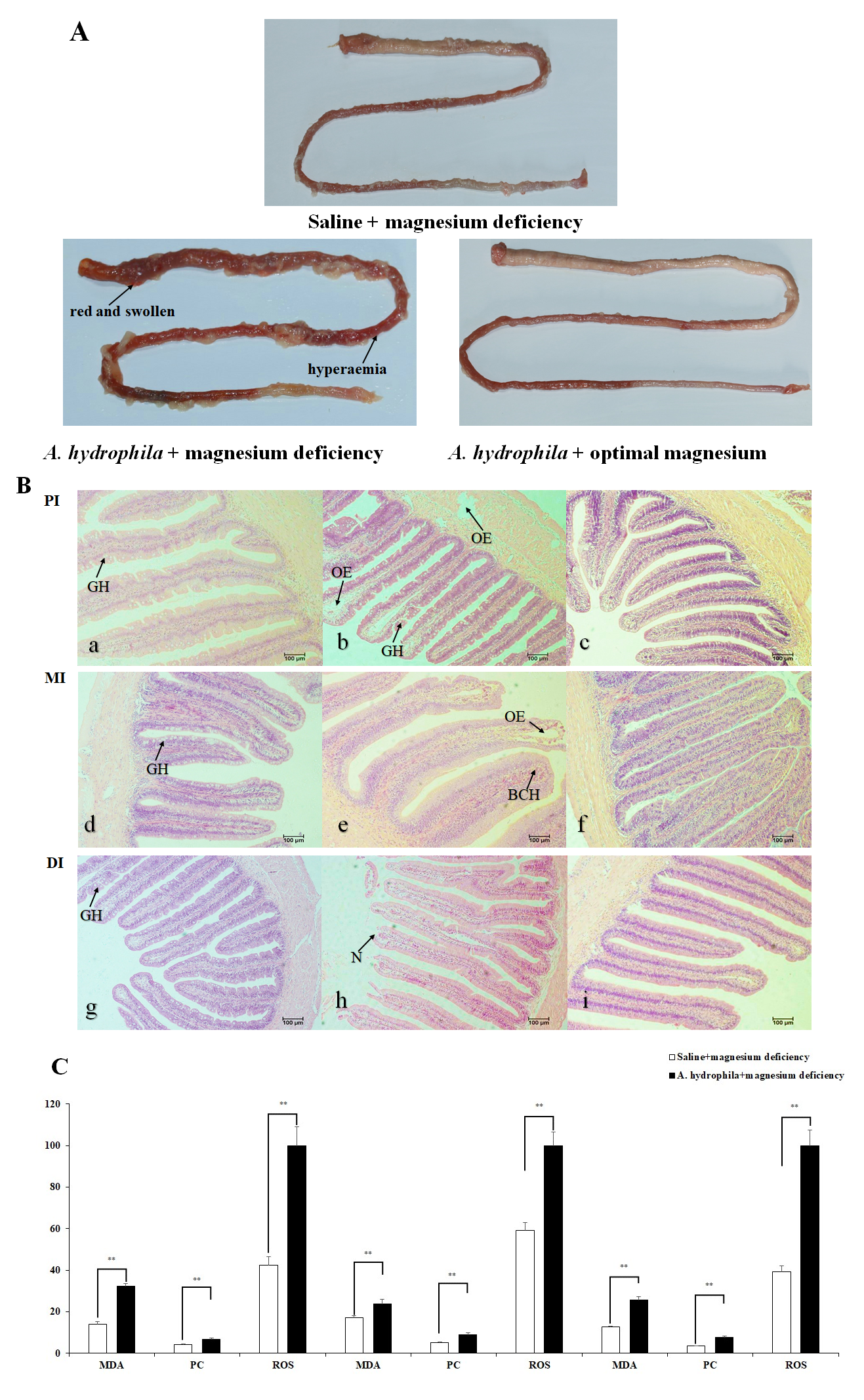


**Figure. S1** Effects of *A. hydrophila* infection on symptom, histology and oxidative damage indices of three intestinal segments of young grass carp (*Ctenopharyngodon idella*) between the saline + magnesium deficiency group*, A. hydrophila* + optimal magnesium group and *A. hydrophila* + magnesium deficiency group. (A) Compared to *A. hydrophila* + optimal magnesium group and saline + magnesium deficiency group, *A. hydrophila* + magnesium deficiency group led to obvious red and swollen as well as hyperaemia in the intestine of young grass carp. (B) Histology of three segments of intestine. The saline + magnesium deficiency group (a, d, g), the *A. hydrophila* + magnesium deficiency group (b, e, h) and the *A. hydrophila* + optimal magnesium group (c, f, i). Arrowhead showed the goblet cell hyperplasia (GH), oedema (OE), blood capillary hyperemia (BCH), necrosis (N). (C) Oxidative damage indices. Magnesium deficiency group: 73.54 mg magnesium kg^-1^ diet group. Optimal magnesium group: 861.67 mg magnesium kg^-1^ diet group.

**Figure. S2**

**PI:**


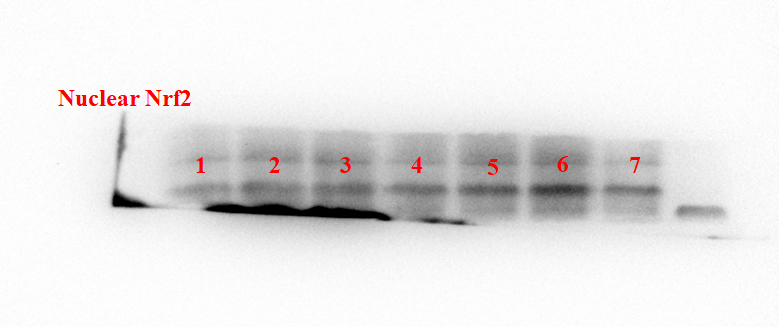


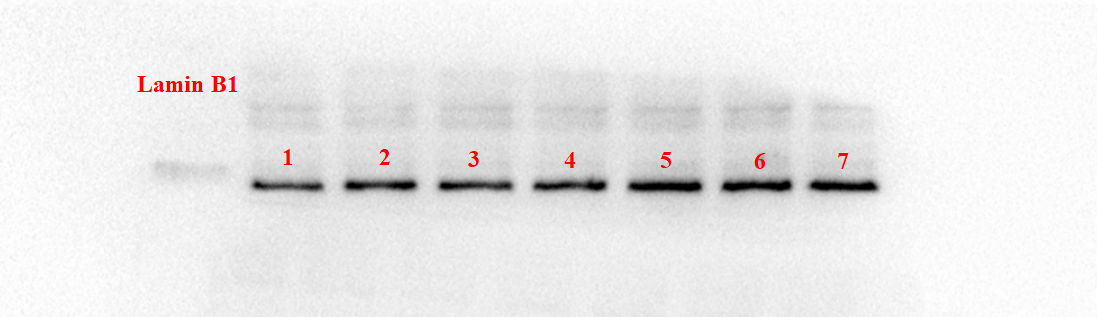

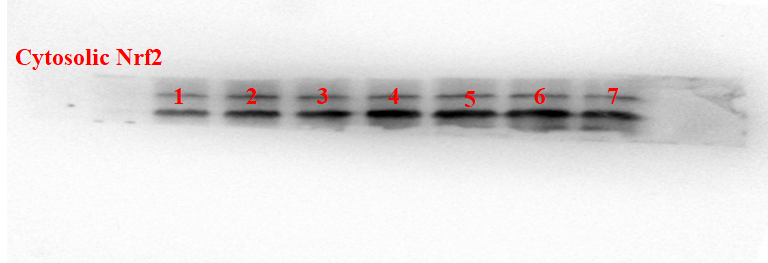


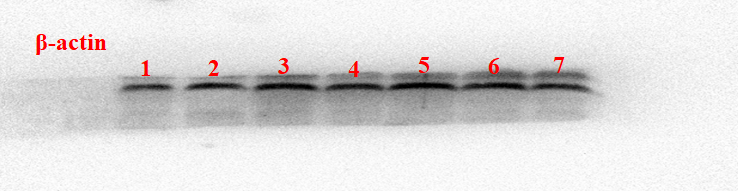


**MI:**


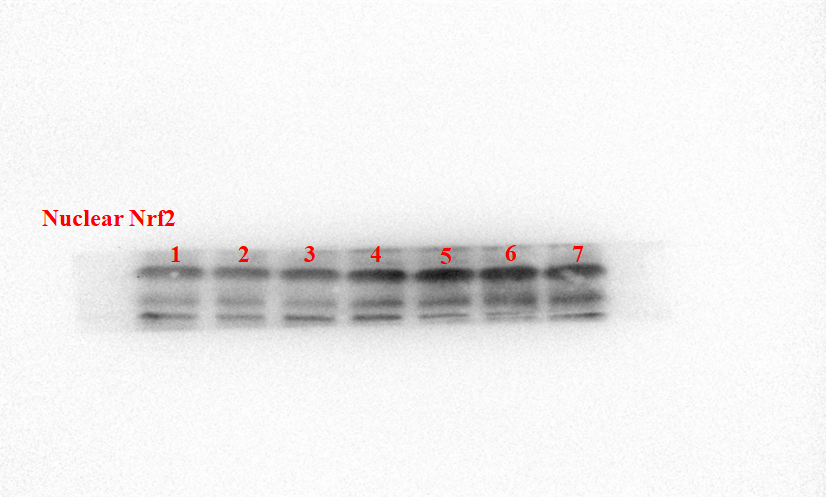


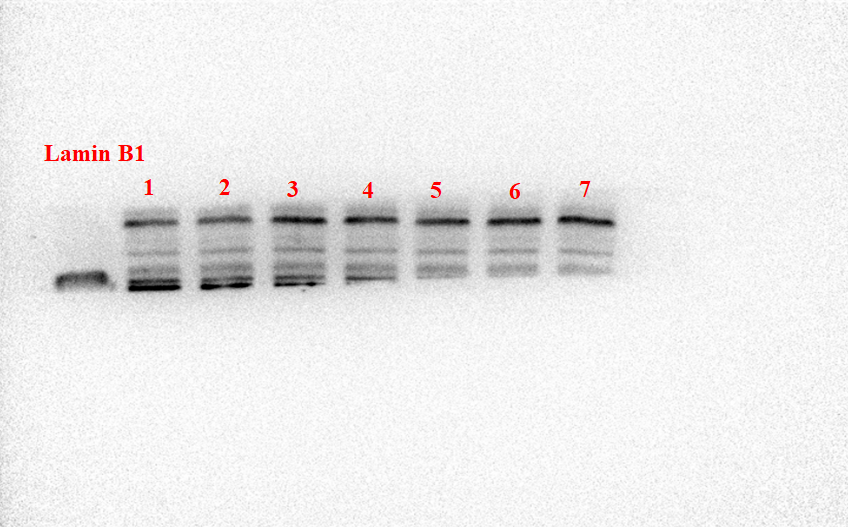

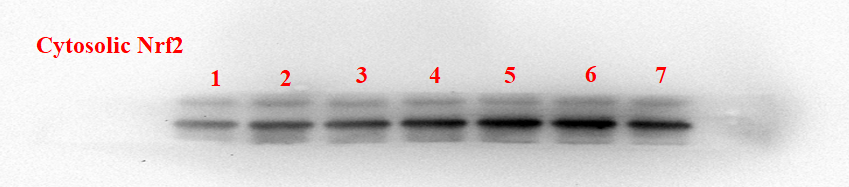


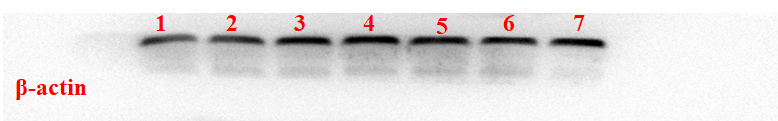


**DI:**


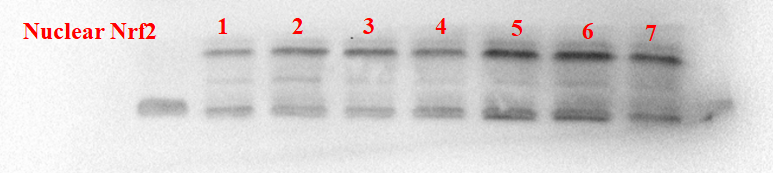


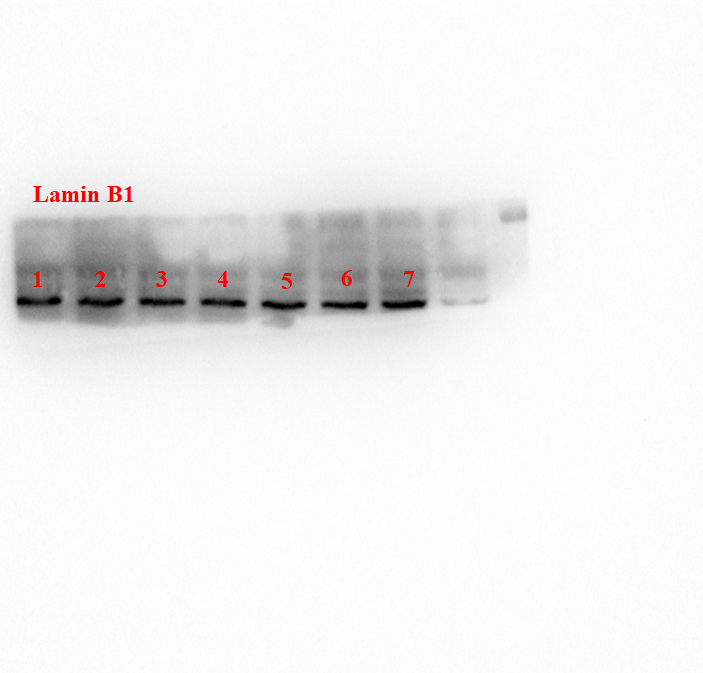


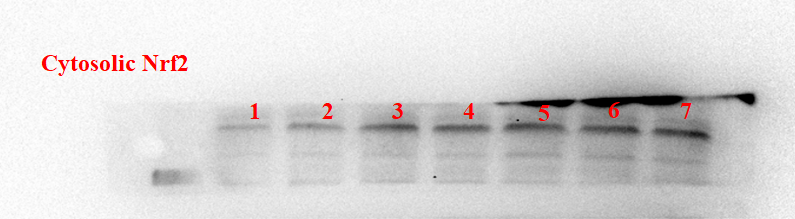


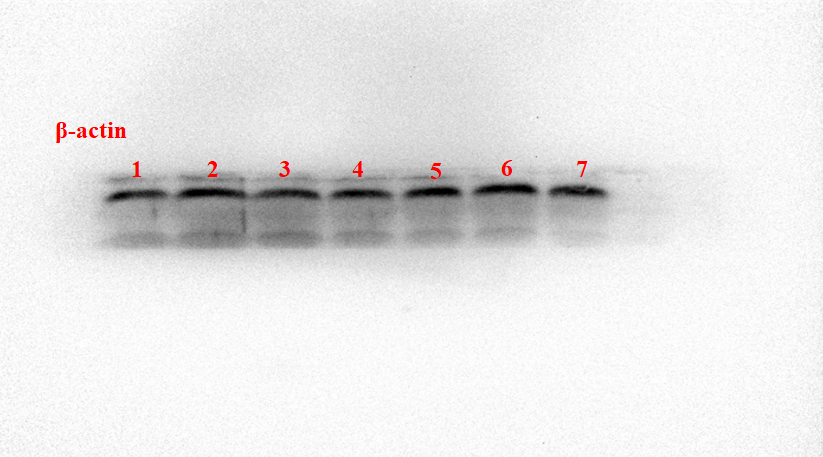


**Figure. S2** Western blot analysis of nuclear Nrf2 and cytosolic Nrf2 in the PI, MI and DI of young grass carp fed diets containing graded levels of magnesium. Lane 1: magnesium deficiency + saline: 73.54 mg kg^-1^ + saline. Lane 2 - Lane 7: levels of dietary magnesium + *A. hydrophila*: 73.54 mg kg^-1^ + *A. hydrophila*, 281.37 mg kg^-1^ + *A. hydrophila*, 487.49 mg kg^-1^ + *A. hydrophila*, 691.55 mg kg^-1^ + *A. hydrophila*, 861.67 mg kg^-1^ + *A. hydrophila* and 1054.53 mg kg^-1^ + *A. hydrophila*, respectively. (We only take the Lane 2 - Lane 7 in the manuscript for analysis).

**Table S1** Real-time primer sequences, thermocycling conditions and accession numbers for 18S rRNA, elongation factor 1 alpha (EF1-α), beta-actin (β-actin) and glycer-aldehyde-3-phosphate dehydrogenase (GAPDH) genes.

| **Target gene** | **Primer sequence** | **Primer sequence** | **Annealing temperature (°C)** | **Accession**  **number** |
| --- | --- | --- | --- | --- |
|  | **Forward (5’ → 3’)** | **Reverse (5’ → 3’)** |  |  |
| ***18S rRNA*** | ATTTCCGACACGGAGAGG | CATGGGTTTAGGATACGCTC | 59.0 | EU047719 |
| ***EF1-α*** | CGCCAGTGTTGCCTTCGT | CGCTCAATCTTCCATCCCTT | 59.0 | GQ266394 |
| ***GAPDH*** | GTTACAAGGGAGAAGTTCACCAT | CCGGTAGACTCGACTACATACAG | 61.4 | GQ266395 |
| ***β-actin*** | GGCTGTGCTGTCCCTGTA | GGGCATAACCCTCGTAGAT | 61.4 | M25013 |

**Table S2** The cycle threshold (Ct) values of each housekeeping gene in the PI, MI and DI of young grass carp fed with graded levels of magnesium (g kg^-1^ diet) *.

| **Magnesium level** | **73.54** | |  | **281.37** | |  | **487.49** | |  | **691.55** | |  | **861.67** | |  | **1054.53** | |
| --- | --- | --- | --- | --- | --- | --- | --- | --- | --- | --- | --- | --- | --- | --- | --- | --- | --- |
| **(mg kg^-1^)** | **Mean** | **SD** |  | **Mean** | **SD** |  | **Mean** | **SD** |  | **Mean** | **SD** |  | **Mean** | **SD** |  | **Mean** | **SD** |
| **PI** | | | | | | | | | | | | | | | | | |
| ***18S rRNA*** | 13.19^a^ | 0.64 |  | 13.23^a^ | 0.17 |  | 13.43^a^ | 0.18 |  | 13.53^a^ | 0.28 |  | 13.63^a^ | 0.25 |  | 13.52^a^ | 0.54 |
| ***EF1-α*** | 22.05^a^ | 1.06 |  | 22.07^a^ | 0.68 |  | 22.23^a^ | 0.36 |  | 22.48^a^ | 0.23 |  | 22.92^a^ | 1.07 |  | 22.87^a^ | 0.71 |
| ***GAPDH*** | 24.14^a^ | 0.40 |  | 24.62^a^ | 0.46 |  | 24.07^a^ | 0.59 |  | 24.39^a^ | 0.62 |  | 23.87^a^ | 0.42 |  | 23.96^a^ | 0.47 |
| ***β-actin*** | 16.05^a^ | 0.13 |  | 16.40^a^ | 0.20 |  | 16.03^a^ | 0.22 |  | 16.17^a^ | 0.52 |  | 16.10^a^ | 0.63 |  | 15.90^a^ | 0.52 |
| **MI** | | | | | | | | | | | | | | | | | |
| ***18S rRNA*** | 13.34^a^ | 0.07 |  | 13.57^a^ | 0.17 |  | 13.54^a^ | 0.35 |  | 13.58^a^ | 0.35 |  | 13.68^a^ | 0.21 |  | 13.49^a^ | 0.48 |
| ***EF1-α*** | 22.51^a^ | 0.65 |  | 22.95^a^ | 0.86 |  | 23.27^a^ | 0.38 |  | 23.23^a^ | 0.71 |  | 22.68^a^ | 0.50 |  | 22.63^a^ | 0.37 |
| ***GAPDH*** | 24.53^a^ | 0.38 |  | 24.19^a^ | 0.52 |  | 24.56^a^ | 0.48 |  | 24.58^a^ | 0.65 |  | 24.88^a^ | 0.52 |  | 24.43^a^ | 0.79 |
| ***β-actin*** | 16.66^a^ | 0.34 |  | 16.74^a^ | 0.35 |  | 16.87^a^ | 0.36 |  | 16.78^a^ | 0.12 |  | 17.02^a^ | 0.15 |  | 16.90^a^ | 0.52 |
| **DI** | | | | | | | | | | | | | | | | | |
| ***18S rRNA*** | 13.67^a^ | 0.37 |  | 13.84^a^ | 0.16 |  | 13.84^a^ | 0.05 |  | 14.07^a^ | 0.48 |  | 13.79^a^ | 0.06 |  | 13.97^a^ | 0.38 |
| ***EF1-α*** | 22.01^a^ | 0.19 |  | 22.56^a^ | 0.46 |  | 22.51^a^ | 0.75 |  | 22.12^a^ | 0.69 |  | 21.93^a^ | 0.20 |  | 21.97^a^ | 0.78 |
| ***GAPDH*** | 24.00^a^ | 0.30 |  | 24.07^a^ | 0.51 |  | 23.87^a^ | 0.66 |  | 23.83^a^ | 0.59 |  | 24.08^a^ | 0.57 |  | 23.90^a^ | 0.58 |
| ***β-actin*** | 16.00^a^ | 0.36 |  | 16.24^a^ | 0.37 |  | 16.17^a^ | 0.35 |  | 16.14^a^ | 0.35 |  | 16.56^a^ | 0.51 |  | 16.27^a^ | 0.40 |

* Values are means and standard deviations of six replicates.

Different superscripts in the same row are significantly different (*P* < 0.05).

**Table S3** Ranking of four selected internal control gene stability values in the PI, MI and DI of young grass carp fed with graded levels of magnesium (g kg^-1^ diet) *.

| **Gene name** | **geNorm M value**  **(order)** | **NormFinder stability**  **value (order)** | **Ranking**  **order** |
| --- | --- | --- | --- |
| **PI** | | | |
| **18S rRNA** | 0.795 (2) | 0.178 (2) | 2 |
| **EF1-α** | 1.012 (4) | 0.267 (4) | 4 |
| **GAPDH** | 0.801 (3) | 0.204 (3) | 3 |
| **β-actin** | 0.772 (1) | 0.172 (1) | 1 |
| **MI** | | | |
| **18S Rrna** | 0.691 (2) | 0.121 (2) | 2 |
| **EF1-α** | 0.805 (4) | 0.173 (4) | 4 |
| **GAPDH** | 0.757 (3) | 0.154 (3) | 3 |
| **β-actin** | 0.680 (1) | 0.117 (1) | 1 |
| **DI** | | | |
| **18S Rrna** | 0.682 (2) | 0.117 (2) | 2 |
| **EF1-α** | 0.769 (4) | 0.173 (4) | 4 |
| **GAPDH** | 0.763 (3) | 0.146 (3) | 3 |
| **β-actin** | 0.661 (1) | 0.087 (1) | 1 |

* The input data for geNorm and NormFinder were 2^-ΔCT^ values.
